# Research on the Recovery and High-Value Utilization Pathways of Valuable Elements from Coal Gangue: A Review

**DOI:** 10.3390/molecules31213944

**Published:** 2026-11-02

**Authors:** Jinxin Zou, Ximei Zhang, Jie Xiong

**Affiliations:** School of Chemical Engineering, Guizhou Minzu University, Guiyang 550025, China

**Keywords:** coal gangue, valuable elements, recovery, extraction, high-value utilization

## Abstract

As the largest solid waste generated by the coal industry, massive stockpiling of coal gangue not only encroaches on land resources but also poses severe ecological and environmental risks. However, coal gangue is rich in recoverable elements: bulk constituents such as aluminum (Al) and iron (Fe), the matrix component silicon (Si), and supply-critical elements such as lithium (Li), gallium (Ga), and rare earth elements (REEs). Although silicon (Si) is the dominant matrix component, it is recovered mainly as a byproduct during the extraction of these metals. Coal gangue holds significant potential as an “urban mine” for resource recovery. In light of this, this paper first elucidates the mineralogical characteristics and physicochemical properties of coal gangue, and reviews pretreatment activation processes such as mechanical and roasting activation aimed at enhancing reactivity. Secondly, it focuses on mainstream technological routes for valuable element extraction, separation and purification, and the high-value utilization of tailings. On this basis, the challenges faced by existing technologies in terms of energy consumption, costs, and secondary pollution are thoroughly analyzed, and future development directions in this field are outlined. Focusing primarily on Chinese coal gangue and targeting Al, Fe, Li, Ga and REEs (with silicon treated as the matrix component and recovered mainly as a byproduct), this review covers the literature published up to July 2026 identified through Web of Science and Scopus. This review aims to provide a scientific reference for establishing a “green extraction–high-value utilization” industrial chain for coal gangue, thereby promoting the green and low-carbon transformation of the coal industry.

## 1. Introduction

**Figure 1:**
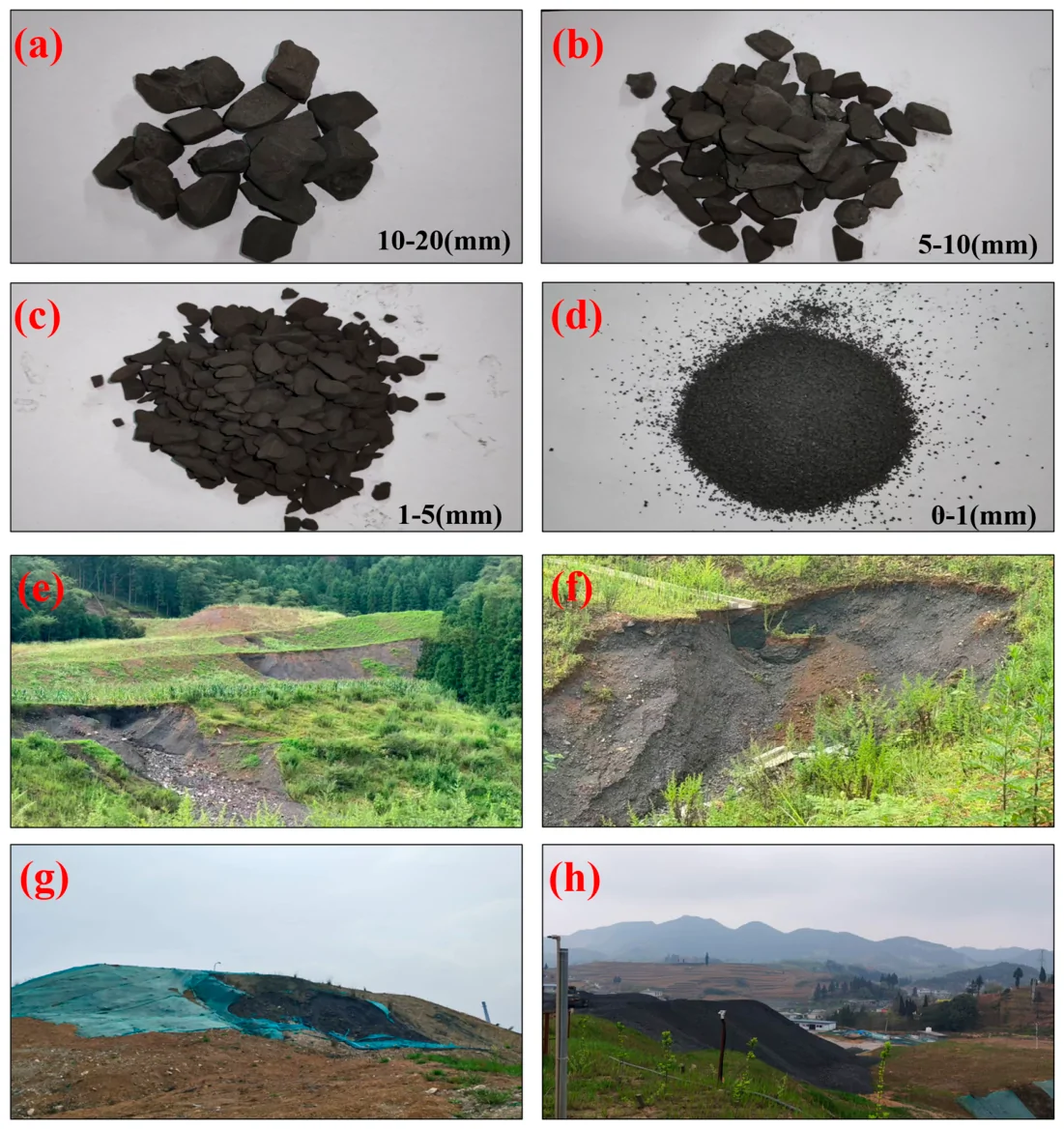
Coal gangue with varying particle sizes: (**a**) 10–20 mm, (**b**) 5–10 mm, (**c**) 1–5 mm, (**d**) 0–1 mm; coal gangue stockpiles: (**e**–**h**) coal gangue stockpiles in open-air dumps.

Coal gangue is the primary solid waste generated during coal mining and washing processes, accounting for approximately 10–20% of raw coal production [1,2]. As a dark gray sedimentary rock, coal gangue is mainly composed of shale, mudstone, sandstone, and residual coal. It is characterized by low carbon content, high hardness, and a density ranging from 1.5 to 2.0 g/cm^3^, and is typically associated with coal seams (Figure 1a–d: Coal gangue with varying particle sizes). Limited by its low calorific value and bottlenecks in traditional utilization technologies, coal gangue has long been regarded as a low-value industrial waste. Massive amounts of coal gangue are disposed of in open-air dumps around mining areas, forming extensive gangue mountains (Figure 1e–h: Coal gangue stockpiles). Taking China as an example, the cumulative stockpile of coal gangue has currently exceeded 7 billion tons [3]. In 2024, China’s raw coal production reached 4.76 billion tons, with associated coal gangue production surpassing 800 million tons [4,5].

For the purposes of this review, coal gangue is operationally defined as the solid rock waste rejected during underground and surface coal mining (excavation rock, roof and floor rock, and partings) and during coal preparation (washing tailings). It is characterized by a low calorific value, a high ash content, and a high content of aluminosilicate minerals such as kaolinite, illite, and quartz. It is distinguished from several related materials: (i) coal refuse is a broader, loosely defined term that may also encompass coal slime and other fine coal-processing wastes; in this review, the term coal gangue is used specifically for the coarser, rock-dominant fraction, and studies that explicitly target coal refuse sensu lato are not treated as coal-gangue studies; (ii) middlings are an intermediate washability product with a recoverable calorific value rather than a waste, and are not considered in this review; (iii) coal-series kaolinite (also termed coal-bearing kaolin or tonstein) is a kaolinite-rich variant of coal gangue that has reached industrial clay grade and is sometimes marketed as a raw material rather than a waste; such industrial-grade coal-series kaolinite is excluded, whereas kaolinite-rich gangue that has not reached industrial clay grade remains within scope; and (iv) other coal-based solid wastes such as fly ash, bottom ash, and coal slime are combustion or fine-grain processing residues with distinct mineralogy and are outside the scope of this review.

Coal-gangue stockpiles are reported to occupy approximately 70 km^2^ of land [3], causing landscape degradation and preventing the use of that land for agriculture and other purposes.

**Figure 2:**
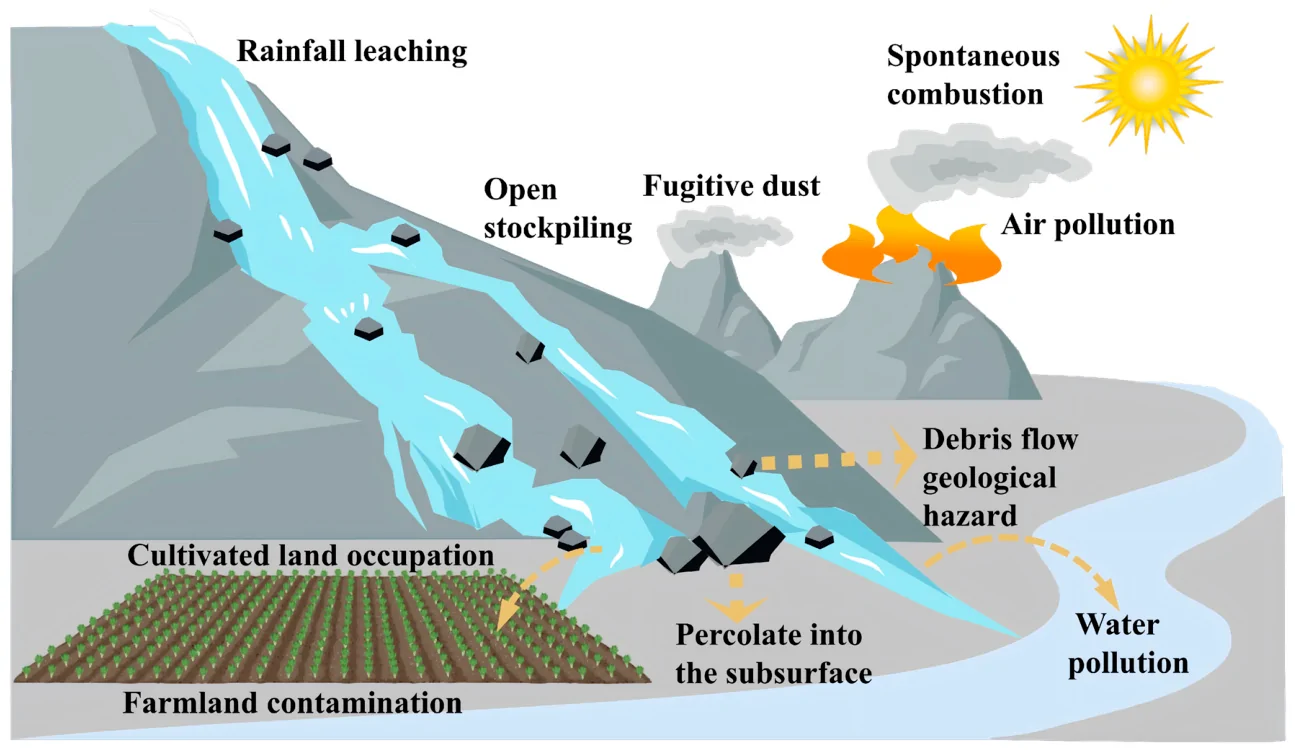
Schematic diagram of the environmental hazards posed by coal gangue.

The massive stockpiling of coal gangue not only encroaches upon valuable land resources but also triggers severe ecological and environmental issues. Coal-gangue stockpiles are reported to occupy approximately 70 km^2^ of land [3]. Due to the residual carbon and pyrite within the gangue, these stockpiles are highly susceptible to spontaneous combustion; in China, about one-third of large-scale gangue dumps have experienced or are currently undergoing self-ignition [3]. This process not only releases heavy metals (e.g., As, Pb) and harmful gases (e.g., CO, CO_2_, H_2_S, SO_2_) into the atmosphere, causing severe air pollution [3], but also induces Acid Mine Drainage (AMD) triggered by spontaneous combustion and pyrite oxidation. The resulting leachate contains significantly elevated concentrations of Fe^2+^, Fe^3+^, SO_4_^2−^, and heavy metals such as Zn, Cu, Cd, Pb, and Ni, posing severe contamination risks to surrounding groundwater and soil [6] (Figure 2). Studies have found that the comprehensive ecological risk index in typical mining areas ranges from 351.51 to 412.27, indicating a high-risk level [3,7]. These environmental hazards underscore the urgency of developing and utilizing coal gangue resources.

Promoting the resource utilization of coal gangue has become an urgent demand for achieving the green transformation of the coal industry. In recent years, research on the utilization of coal gangue in co-firing for power generation, building material preparation, underground backfilling, ecological restoration, soil amendment, and CO_2_ mineralization sequestration has continued to deepen, with continuous technological breakthroughs [8,9,10,11,12]. Recent work has also demonstrated additional pathways for converting coal-related waste into valuable products: Lysyi et al. [13] analyzed the thermodynamic feasibility of coal-mining-waste gasification for hydrogen-rich synthesis gas, while Kuanganov and Fischer [14] reviewed coal-based germanium production from Kazakhstan coals, covering Ge extraction from coal feedstock and processing byproducts. However, the large-scale promotion of these technologies is still constrained by dual bottlenecks in techno-economic feasibility and engineering adaptability [9].

**Figure 3:**
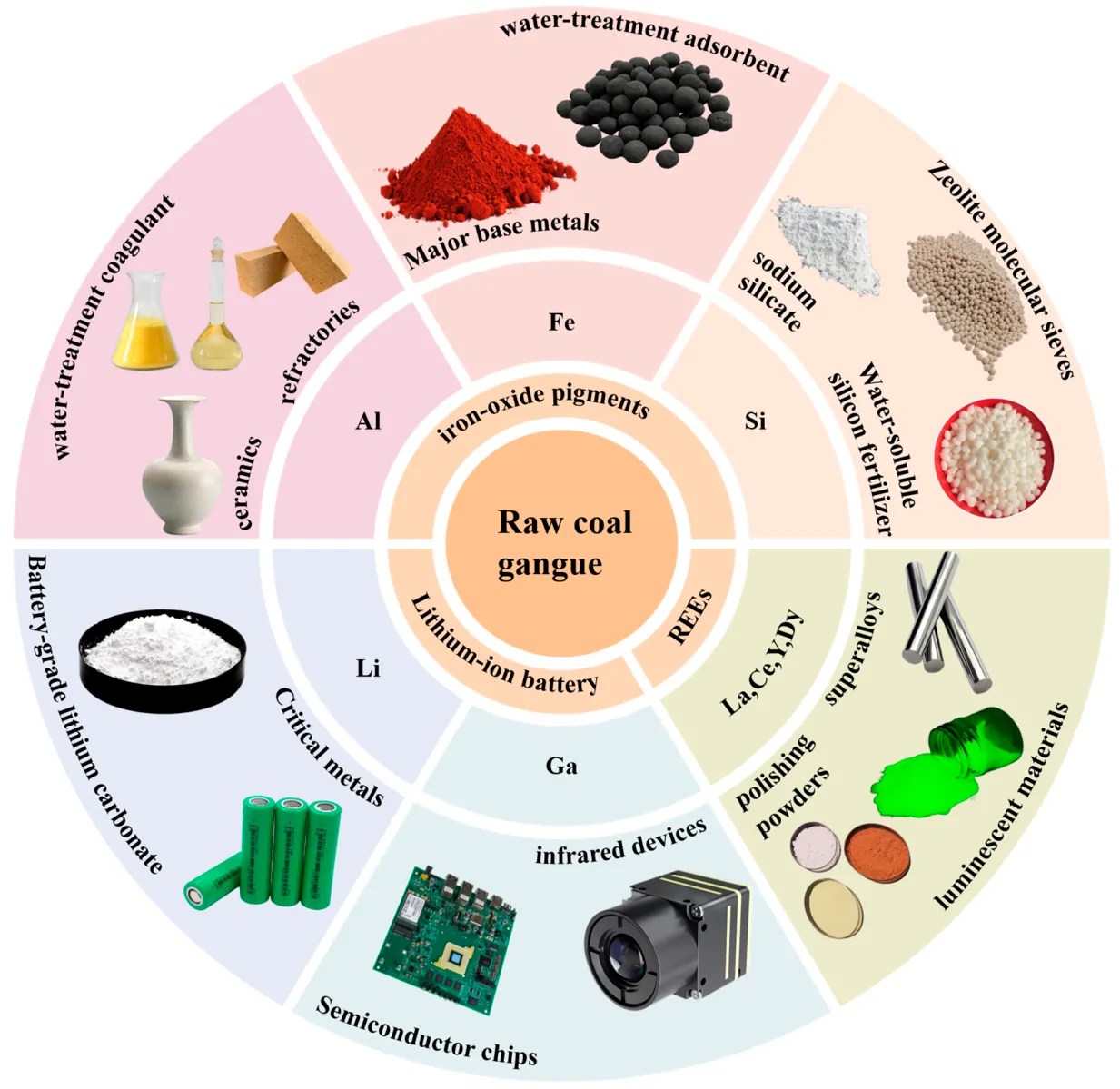
Applications of aluminum (Al), iron (Fe), lithium (Li), gallium (Ga), and rare earth elements (REEs) in industrial production.

Coal gangue is enriched with critical elements such as aluminum (Al), iron (Fe), lithium (Li), gallium (Ga), and rare earth elements (REEs). As the cornerstone of industrial production, these metallic elements play a pivotal role in the national economy (Figure 3). Against the macro backdrop of tightening global supplies of critical minerals, the efficient recovery and high-value utilization of these key metals from coal gangue have emerged as a core hotspot and frontier direction in the field of comprehensive coal gangue utilization [15,16]. After years of exploration, although significant progress has been made in this field, the following issues remain: First, coal gangue typically exhibits a highly ordered crystal structure and exceptional chemical stability. This “inert” characteristic makes it difficult to dissociate the valuable elements hosted within, resulting in extremely low extraction efficiencies via direct acid leaching or alkaline dissolution [17], thus usually requiring pre-activation treatment. Second, these valuable elements are characterized by diverse occurrence forms and complex associations [18]. This multi-element coexistence and multi-phase coupling dictate that the design of extraction processes must be highly targeted. Third, the complex enrichment and purification processes applied to the leachates consume substantial amounts of acids, alkalis, and organic extractants. Without closed-loop management, these processes are prone to generating high-acidity wastewater, volatile exhaust gases, and refractory secondary solid wastes, leading to severe “pollution transfer.” Therefore, the greatest challenge facing this field remains how to design selective structural disruption pathways based on the occurrence mechanisms of elements within the inert aluminosilicate matrix, achieving efficient stepwise release and recovery of target elements while maximizing economic benefits, and simultaneously addressing environmental pollution throughout the entire process.

Compared with previous coal-gangue reviews that focused on either single-element extraction (e.g., alumina from kaolinitic gangue) or on building-material applications [8,10], the present review differs in three respects: (1) it covers the full process chain from mineralogical characterization through activation, leaching, separation/purification, and final-product valorization for five target elements (Al, Fe, Li, Ga, REEs) [16]; (2) it evaluates activation and leaching routes against consistent methodological criteria—including activation–leaching selectivity, downstream purification burden, and environmental performance—rather than reporting maximum extraction percentages in isolation [9,15]; and (3) it explicitly treats integrated multi-element (synergistic) recovery as a central review question, identifying the scarcity of complete flowsheet-level data as a key research gap [8,9,15].

Accordingly, this review is organized around three questions: (1) which pretreatment activation strategy most effectively disrupts the inert aluminosilicate matrix and improves element leachability, and how mineralogical controls (occurrence modes and mineral association) govern activation–leaching selectivity; (2) which techno-economic and environmental bottlenecks limit industrial scale-up of existing extraction routes, covering downstream separation/purification, environmental performance (secondary pollution and reagent/energy consumption), and scale-up feasibility; and (3) how multi-element co-extraction can be designed to achieve synergistic recovery while minimizing waste and secondary pollution. Across these questions, the review evaluates five interrelated dimensions: mineralogical controls on element release, activation–leaching selectivity, downstream separation and recovery, environmental performance, and scale-up feasibility. Briefly, the boundaries are: feedstocks limited to Chinese coal gangue (excluding coal refuse sensu lato, fly ash, bottom ash, coal slime, middlings, and coal-series kaolinite of industrial clay grade); target elements Al, Fe, Li, Ga and REEs (Si as byproduct); process stages from pretreatment to recovered-product valorization (excluding combustion, building materials, backfilling, ecological restoration, and cementitious alkali activation/geopolymerization); products limited to metals and their compounds (excluding bulk construction materials); and technology readiness weighted toward laboratory-scale hydrometallurgical studies.

This review aims to provide a scientific reference for establishing a “green extraction–high-value utilization” industrial chain for coal gangue.

## 2. Materials and Methods

This review follows a narrative design; nevertheless, the search and selection procedure is documented below for transparency. The literature search was conducted between April and July 2026 using two bibliographic databases—Web of Science and Scopus—supplemented by targeted hand-searching of Chinese dissertations (CNKI, Wanfang), statistical yearbooks, and institutional reports, which were included only when they contained primary quantitative data unavailable in the peer-reviewed literature. Search strings combined three blocks: feedstock (“coal gangue”), target elements (Al, Fe, Li, Ga, REEs), and process (“leaching”, “extraction”, “recovery”, “activation”).

Records were deduplicated, screened by title/abstract, then assessed at full text against the following criteria. Inclusion: primary studies reporting quantitative recovery data (with denominator stated) for at least one target element from coal gangue; English or Chinese language; published from 2000 onward. Exclusion: non-gangue feedstocks (e.g., coal refuse sensu lato, fly ash, bottom ash, coal slime, middlings, and coal-series kaolinite of industrial clay grade); non-element-recovery processes (e.g., combustion, building materials, backfilling, ecological restoration); reviews or editorials without primary data; and duplicate datasets. Novelty was not used as an eligibility criterion; confirmatory or negative studies were retained.

**Figure 4:**
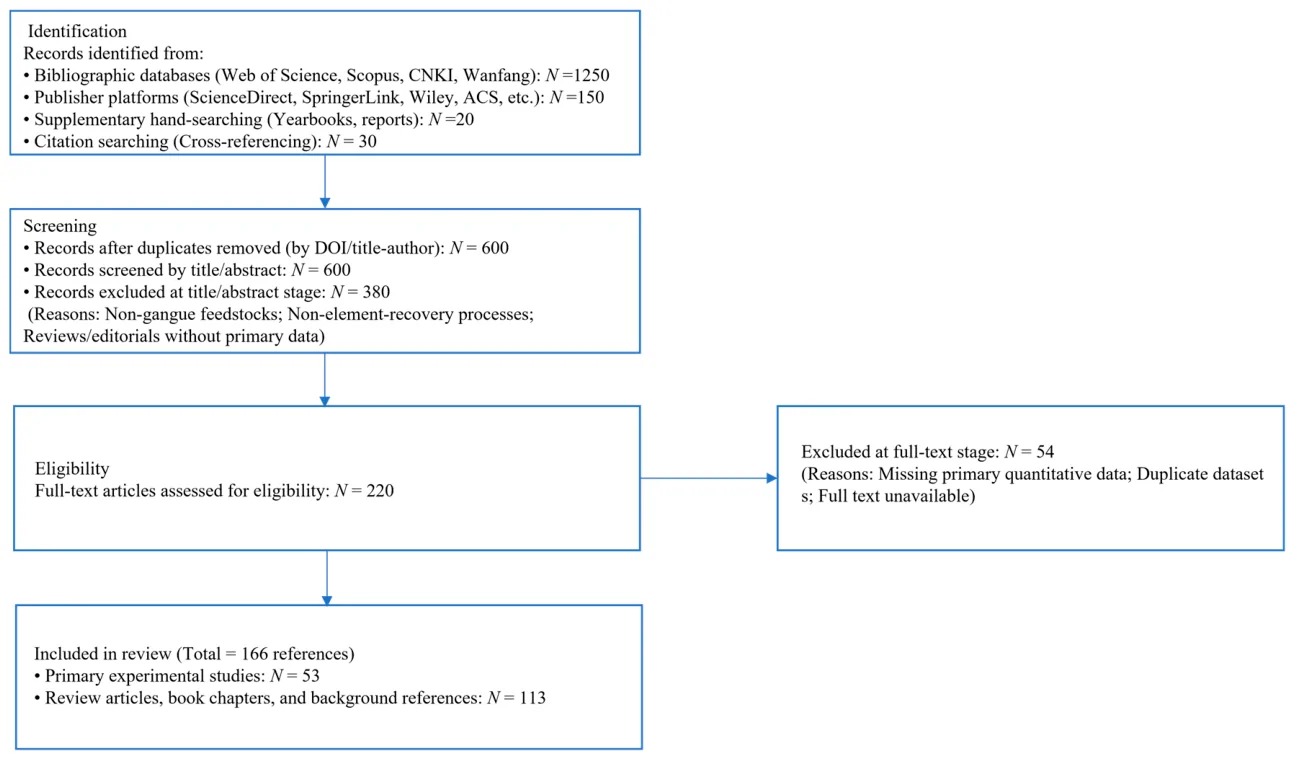
PRISMA-style flow diagram of the literature search and screening process. A total of 1450 records were initially identified from Web of Science, Scopus, publisher platforms, and supplementary hand-searching. After removing duplicates (*n* = 850), 600 records were screened by title and abstract, resulting in 220 records for full-text assessment. After excluding 54 full-text records based on the predefined inclusion/exclusion criteria, the final corpus comprised 166 references, including 53 primary experimental studies and 113 review/background references.

Two authors independently performed screening, with disagreements resolved by discussion with the third. A total of 1450 records were initially identified. Following a rigorous screening process (including deduplication and full-text assessment), 166 references were ultimately included in the final corpus, comprising 53 primary experimental studies and 113 review/background references. The detailed literature search and screening process is summarized in Figure 4.

## 3. Mineralogical Characteristics and Physicochemical Properties of Coal Gangue

### 3.1. Mineralogical and Chemical Compositions in Coal Gangue

**Figure 5:**
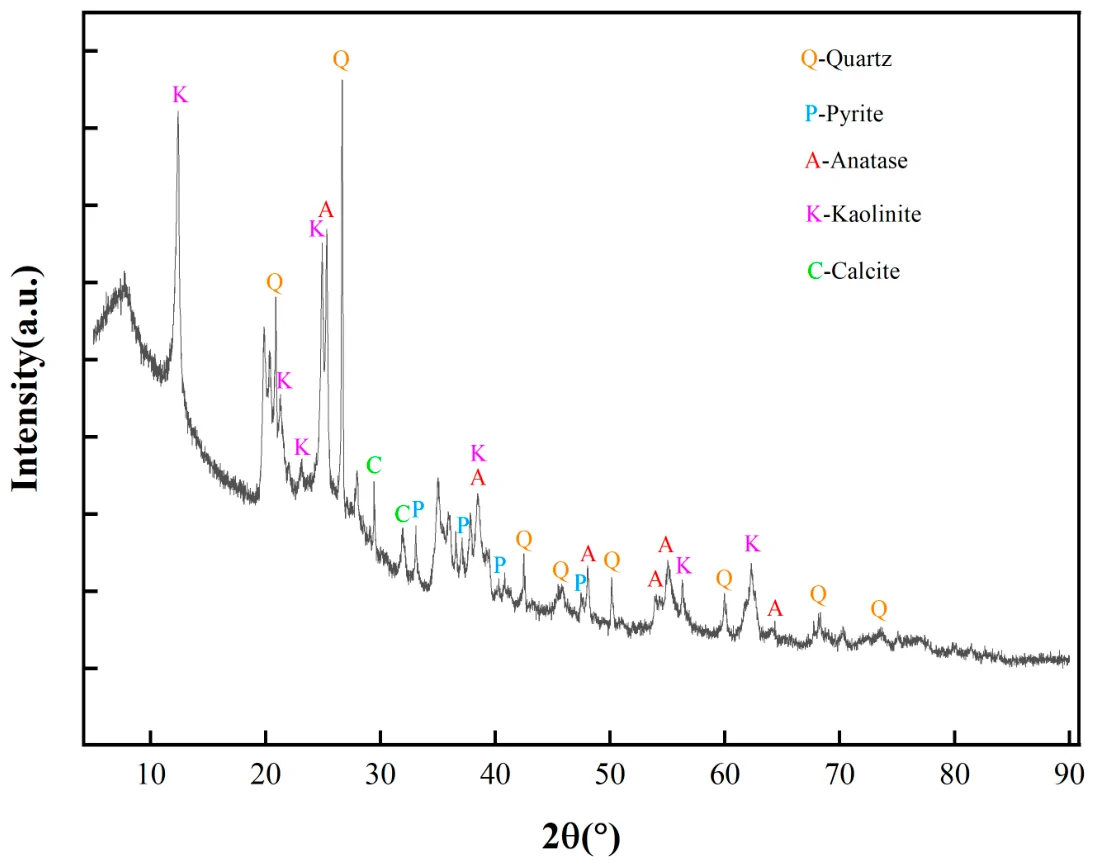
XRD patterns of a representative coal gangue sample from Guizhou Province, China. The main mineral phases (quartz, pyrite, kaolinite, calcite, and anatase) are labeled.

**Figure 6:**
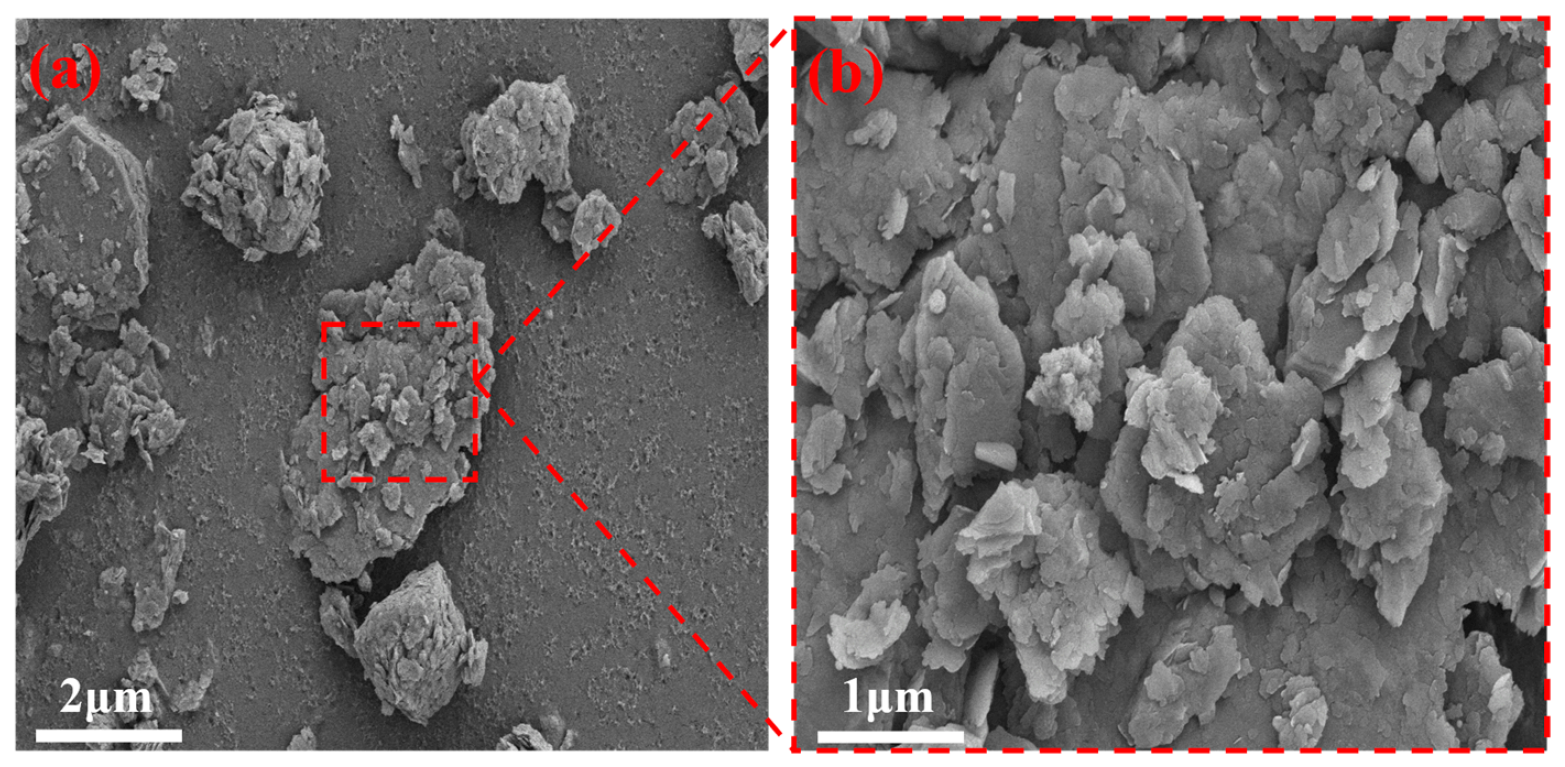
SEM images of the coal gangue sample: (**a**) overall particle morphology; (**b**) magnified view of the red dashed region in (**a**), showing the compacted, lamellar structure.

Kaolinite (Al_2_Si_2_O_5_(OH)_4_) is a common major clay mineral in many coal gangues, typically occurring together with quartz, illite, montmorillonite, pyrite, siderite, calcite, dolomite, boehmite, and carbonaceous matter, although the relative proportions vary substantially with the geologic setting and coal rank [1] (Figure 5: XRD patterns and Figure 6a,b: SEM images of coal gangue from Guizhou, China). Additionally, trace amounts of ferrosilite (FeSiO_3_), siderite (FeCO_3_), and rutile (TiO_2_) have been identified in coal gangue from certain mining areas [19].

Chemically, coal gangue mainly consists of silicon dioxide (SiO_2_), aluminum oxide (Al_2_O_3_), iron oxide (Fe_2_O_3_), sulfur trioxide (SO_3_), calcium oxide (CaO), magnesium oxide (MgO), potassium oxide (K_2_O), sodium oxide (Na_2_O), and carbon (C). Among them, SiO_2_ and Al_2_O_3_ are the dominant oxides in coal gangue, with typical mass fractions of 35–55% and 15–35%, respectively, accounting for a combined total of 55–85% [20]. Iron in coal gangue primarily exists in the form of pyrite and hematite, with the total iron content (expressed as Fe_2_O_3_) ranging from approximately 1% to 15% [21,22]. Furthermore, the chemical composition of coal gangue exhibits significant regional variations, with specific contents fluctuating depending on the geological conditions of the coal seam and the type of gangue [1,9]. Table 1 summarizes the chemical composition (wt.%, determined by XRF as reported in the original studies) of coal gangue from different regions in China.

### 3.2. Critical Trace Elements in Coal Gangue

In addition to the aforementioned conventional elements, coal gangue is also associated with critical trace elements such as lithium (Li), gallium (Ga), and rare earth elements (REEs). The presence of these critical trace elements provide immense potential for the high-value development and utilization of coal gangue.

#### 3.2.1. Lithium in Coal Gangue

The global average lithium content in coal is 12 μg/g [27]. In contrast, the average lithium content in Chinese coal is 31.8 μg/g [28], which is significantly higher than the global average. In China, the lithium content in coal gangue is generally much higher than that in raw coal. The existing literature indicates that the lithium content in coal gangue generally exceeds 100 μg/g, with a maximum value reaching 550 μg/g (Table 2).

Lithium in coal gangue is typically chemically bonded with silicate minerals [34], primarily adsorbed on the surfaces of clay minerals or incorporated into the crystal lattices of minerals such as kaolinite [35], illite [36,37], and montmorillonite [38]. Multiple sequential chemical extraction studies have consistently demonstrated that the vast majority of lithium (91–98.9%) in coal gangue exists in the residual phase (i.e., aluminosilicate mineral phase). Lithium associated with Fe-Mn oxides, organic matter, and carbonates is present only in trace amounts [39,40]. Furthermore, the lithium content in cryptocrystalline kaolinite is generally higher than that in crystalline kaolinite [32,41].

#### 3.2.2. Gallium in Coal Gangue

Chinese coals generally contain significant levels of Ga (2–20 μg/g) and exhibit anomalous enrichment in specific regions [42]. The Ga content in certain coalfields exceeds 30 μg/g [43,44,45]. The Ga content in coal gangue is comparable to that in raw coal [46]. Table 3 summarizes the Ga concentrations and major Ga-bearing phases in selected Chinese coal gangue samples. Due to the similar ionic radii of Ga and Al, isomorphous substitution between them occurs readily. Consequently, Ga in coal gangue is predominantly hosted in kaolinite, boehmite, and various aluminosilicate clay minerals [47,48,49], occurring either via surface adsorption or by substituting cations within the mineral lattice [50,51,52,53].

#### 3.2.3. Rare Earth Elements in Coal Gangue

Statistical estimates indicate that global coal deposits contain approximately 50 million tonnes of rare earth elements (REEs) as a geologic resource estimate, nearly half of those found in conventional rare earth deposits [55,56,57]. The REE content in coal exhibits significant regional variations, with a global average of approximately 60.07 μg/g [27]. In comparison, REE concentrations vary substantially across different countries: the average content in U.S. coals is 66.29 μg/g [58], while systematically Chinese coals average 140.27 μg/g [59]. Notably, coals from the Russian Far East exhibit exceptionally high concentrations ranging from 500 to 1500 μg/g [60]. An analysis of 696 coal samples collected from Guizhou Province revealed rare earth element and yttrium (REY) contents ranging from 28.95 to 978.19 μg/g, with an average of 154.83 μg/g [61]. The rare earth element (REE) content in coal gangue is slightly higher than that in coal (Table 4 presents the REE content data for selected Chinese coal gangue samples) [54,62,63,64,65,66]. Therefore, coal and coal-bearing strata are considered potential resources for future REE extraction [67,68,69].

REEs in coal gangue are predominantly enriched in kaolinite [63,65]. Their modes of occurrence exhibit significant variations depending on coal types and geographical regions. The primary occurrence forms include independent mineral phases (e.g., phosphate minerals [70]), ion-exchangeable forms, surface adsorption on clay minerals, and chemical bonding with organic matter or functional groups within minerals [28,56,57,71,72,73]. Wu et al. [64] investigated the distribution of REEs across six chemical fractions in coal gangue samples from three different power plants in China. Their results indicated that REEs exist primarily in acid-soluble and silicate-aluminosilicate forms, followed by sulfide and organic forms, while water-soluble and exchangeable fractions account for negligible proportions. This limited migration and transformation potential in the environment is the primary reason for the stable occurrence of REEs in coal gangue.

## 4. Activation Pretreatment Technologies for Coal Gangue

**Figure 7:**
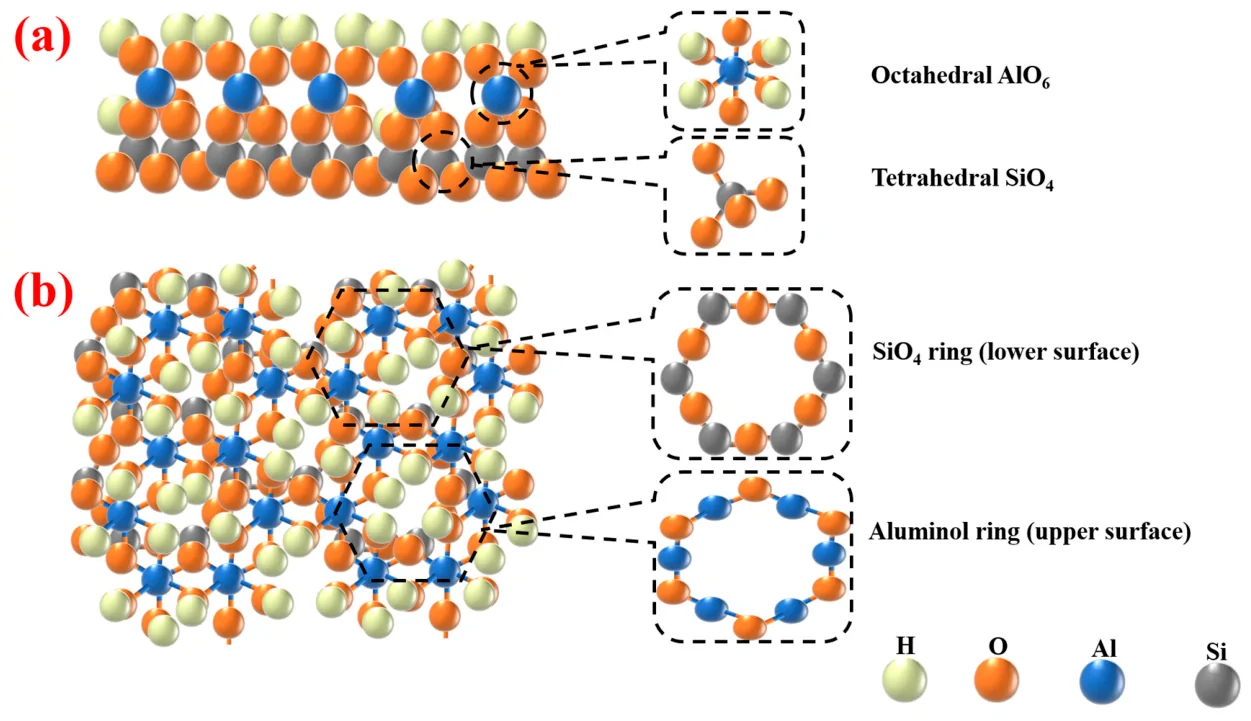
Schematic diagram of the crystal structure of kaolinite (**a**) front view and (**b**) top view.

The primary mineral constituent of coal gangue is kaolinite, which serves as the main source of its alumina and silica components. Kaolinite is a typical 1:1 type phyllosilicate clay mineral composed of alternating SiO_4_ tetrahedral sheets and AlO_6_ octahedral sheets [74] (Figure 7). The tetrahedral sheet consists of corner-sharing SiO_4_ tetrahedra, whereas the octahedral sheet consists of edge-sharing Al-centered AlO_6_ octahedra. Within the tetrahedral sheet, SiO_4_ tetrahedra are arranged in a hexagonal network by sharing basal oxygen atoms, which connect to the adjacent aluminous octahedral sheet [75]. The octahedral sheet is linked to the tetrahedral sheet via shared oxygen atoms, extending along the a- and b-axes and stacking along the c-axis. In the alumina octahedron, each aluminum atom is coordinated by six oxygen atoms: four surface hydroxyl oxygens and two bridging oxygens. The surface hydroxyl oxygens are shared with adjacent aluminum atoms, while the bridging oxygens are shared with silicon atoms [76]. The SiO_4_ tetrahedral and AlO_6_ octahedral sheets are held together by hydrogen bonds between the basal oxygens of the tetrahedral sheet and the hydroxyl groups on the octahedral-sheet surface. Consequently, kaolinite contains two distinct types of hydroxyl groups [75]: (i) outer hydroxyls (also known as inner-surface hydroxyls), located on the external surface of the octahedral sheet with unshared coordination planes, and (ii) inner hydroxyls, situated within the 1:1 structural layer, sharing their coordination planes with the apical oxygen atoms of the tetrahedral sheet. The presence of these structural hydroxyls significantly reduces the chemical reactivity of kaolinite [77]; however, their removal through pretreatment can substantially enhance its reactivity [78].

**Figure 8:**
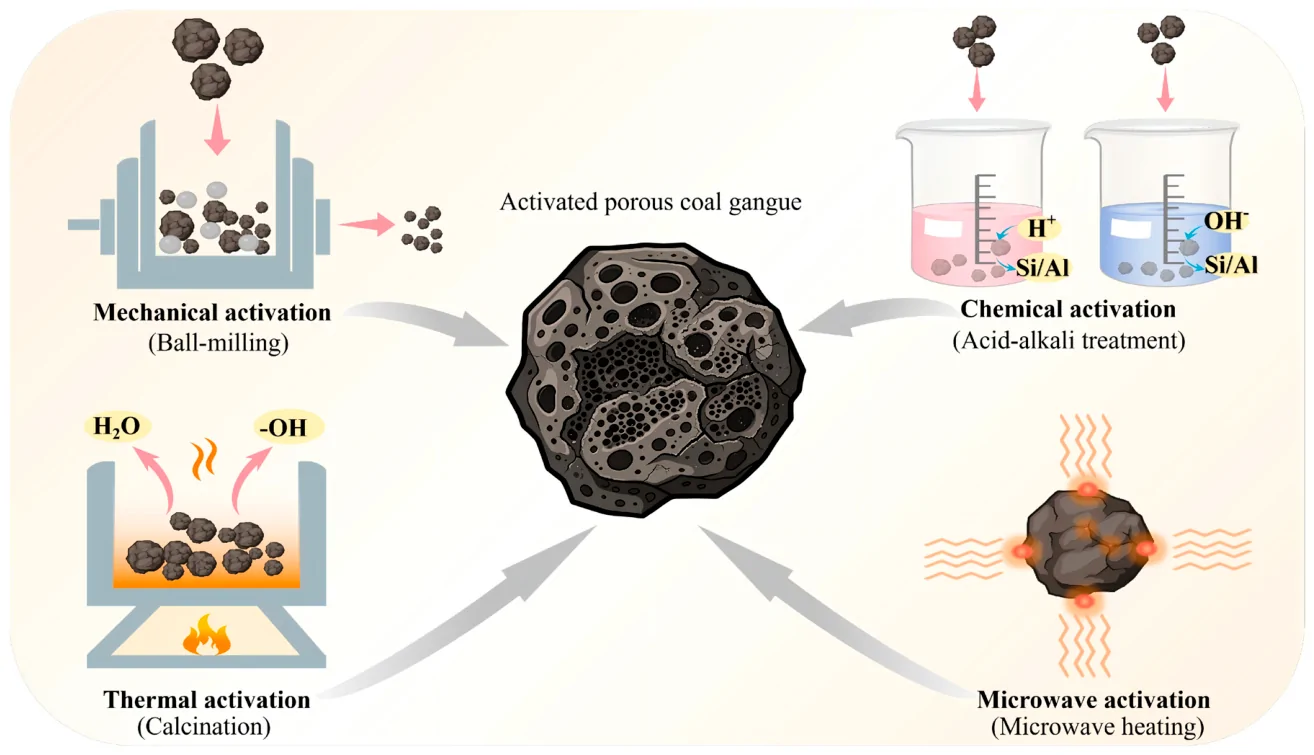
Different activation pretreatment technologies.

Activation pretreatment is a critical step in transforming coal gangue from inert solid waste into an active resource. Fundamentally, this process involves external energy input to disrupt the stable crystal structures of minerals such as kaolinite and quartz, thereby stimulating and releasing active aluminosilicate components to establish a reaction basis for subsequent high-value utilization [3]. Based on the intensity of the applied energy and the degree of reactivity enhancement, existing activation technologies can be categorized into five types: mechanical activation, thermal activation, chemical activation, microwave activation, and combined activation. This classification forms a progressive technological chain, evolving from physical to chemical modification, and from single to synergistic mechanisms (Figure 8).

### 4.1. Mechanical Activation

Mechanical activation refers to a treatment process that utilizes mechanical forces, such as grinding, to induce physicochemical changes in coal gangue [21,79]. The grinding process of coal gangue can generally be divided into three stages: initial fragmentation, particle agglomeration, and agglomerate disaggregation [80]. During this process, the particle size exhibits an evolutionary trend of initially decreasing, followed by a slight increase, and then decreasing again. Grinding not only effectively refines the particle size of coal gangue but also significantly increases its specific surface area and pore volume [80]. Meanwhile, with the increase in specific surface area, the number of effective active sites on the particle surfaces also increases correspondingly [20].

Significant changes in mineral composition, chemical structure, and chemical properties occur in coal gangue during the grinding process. The originally chemically inert kaolinite undergoes lattice disorder, defect formation, particle-size reduction, and partial amorphization under mechanical stress [81,82]. This mechanically induced amorphization should be distinguished from the thermally induced dehydroxylation that converts kaolinite to metakaolin at 400–600 °C; grinding alone does not achieve complete dehydroxylation or full metakaolin formation without direct thermal evidence. Concurrently, spectroscopic and XRD evidence indicates that grinding distorts the local Si–O–Al bond environment and reduces crystallinity [80]; this bond distortion and defect formation lower the activation barrier for subsequent acid or alkaline dissolution, but should be distinguished from actual Si–O and Al–O bond cleavage, which occurs only during the subsequent leaching step. Kristóf et al. [83] suggest that grinding can even lead to the complete destruction of the kaolinite crystal structure. These microstructural alterations significantly improve the thermal decomposition performance of organic matter, kaolinite, and other aluminosilicate minerals in coal gangue. Further studies have revealed that grinding promotes surface aluminum enrichment, increasing the surface aluminum content and exposing new active surfaces [80]. Torres Sánchez et al. [84] and Sun et al. [85] attribute this aluminum enrichment to the formation of an alumina coating induced by the grinding process. In contrast, Fernandez-Bertran [86] posits that mechanical treatment induces structural disorder, thereby generating micro-cracks and new surfaces.

The relationship between grinding duration and the enhancement of reactivity exhibits a distinct non-linear evolution pattern: reactivity increases rapidly during the initial stage of grinding. Guo et al. [80] demonstrated that the leaching rate of alumina increases continuously with grinding time, reaching 70% after 20 h. Wang et al. [87] further pointed out that after 10 h of ball milling, the alumina leaching efficiency increased from 7% to 88%. However, once the particle fineness reaches a certain critical threshold, the rate of reactivity enhancement significantly slows down [20]. This is primarily attributed to the fact that excessive grinding induces particle agglomeration, causing the growth of reactivity to gradually plateau [21].

From a process perspective, mechanical activation offers advantages such as operational simplicity, the absence of high-temperature calcination, and no chemical reagent consumption, making it ecologically safe [21]. However, there is a physical limit to the extent to which this method can disrupt the crystal structure of kaolinite. Prolonged grinding only induces structural deformation in kaolinite and fails to completely disintegrate its Si–O–Al VI layered framework; consequently, the reactivity enhancement achieved by mechanical activation alone is limited [21].

### 4.2. Thermal Activation

As the dominant mineral in coal gangue, kaolinite is a 1:1-type TO phyllosilicate whose Si–O tetrahedral and Al–O octahedral networks are exceptionally stable at ambient temperature. Thermal activation (calcination) reduces its crystallinity and disrupts the interlayer hydrogen bonds through dehydroxylation, promoting the formation of reactive amorphous aluminosilicates [11,88].

**Figure 9:**
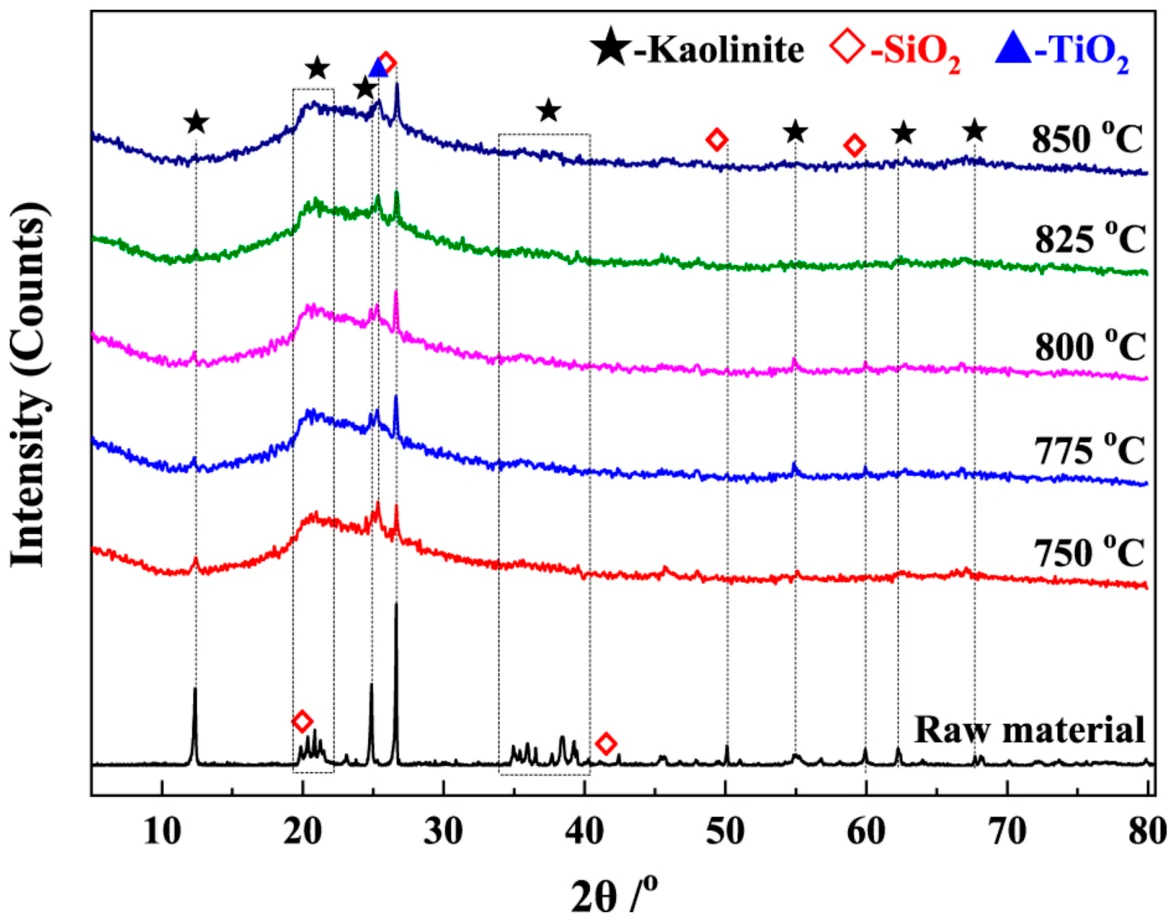
XRD patterns of raw high-alumina coal gangue (Fushun, China) and suspension-calcined products (5 s) at 750, 775, 800, 825, and 850 °C. Kaolinite peaks progressively disappear while quartz and TiO_2_ (internal standard) persist. Adapted from Jiu et al. [89], © 2022, published by Frontiers under CC BY 4.0.

Thermogravimetric (TG) analysis of coal gangue in an air atmosphere identifies four chemically distinct stages of weight loss: (i) removal of adsorbed and interlayer water below 200 °C; (ii) dehydroxylation of kaolinite, spanning approximately 400–700 °C with the endothermic minimum centered near 550 °C; (iii) decomposition of carbonates around 600–750 °C; and (iv) combustion of residual carbon [44,89]. The corresponding phase evolution has been followed by temperature-resolved XRD (Figure 9). At low temperatures, kaolinite remains the dominant crystalline phase, with only minor pyrite beginning to oxidize toward hematite Equation (1). As dehydroxylation proceeds, the characteristic kaolinite reflections at 12.46°, 24.99°, and 38.50° weaken and finally disappear above 750–800 °C, whereas the quartz reflections at 20.9° and 26.7° remain essentially unchanged and an inert TiO_2_ internal standard peak at 25.3° persists throughout the series—confirming that the disappearance of kaolinite peaks reflects a true phase transformation to amorphous metakaolin rather than a mere loss of diffraction intensity or any newly crystallized phase Equation (2) [89,90]. Above 850 °C, the amorphous metakaolin structure is disrupted and solid-state reactions between quartz and alumina generate mullite Equation (3), with trace rutile also appearing [18]. It should be emphasized that the optimal activation temperature is not a fixed universal value, but rather a feedstock- and process-dependent operative window. The dehydroxylation interval shifts considerably with kaolinite crystallinity, heating rate, holding time, particle size, atmosphere, and the presence of additives. Therefore, the commonly cited 600–800 °C range should be interpreted as a typical operative window for conventional thermal activation, rather than a universal optimum [89,90].

These phase assignments are corroborated not only by XRD but also by complementary thermal and vibrational evidence. The endothermic dehydroxylation event in the 550–700 °C interval, together with the progressive loss of inner-surface hydroxyl bands near 3695 and 3670 cm^−1^ and the disappearance of the Si–O–Al (VI) bending bands at 538–550 cm^−1^, independently confirms conversion to metakaolin [89,90]. In addition, boehmite reflections are reported to vanish by 500 °C, after which calcite decomposes near 600 °C and the resulting CaO partially converts to anhydrite [18]. Kong et al. [91] further observed that between 900 and 1000 °C metakaolin undergoes endothermic decomposition toward spinel and amorphous silica Equation (4).

**Figure 10:**
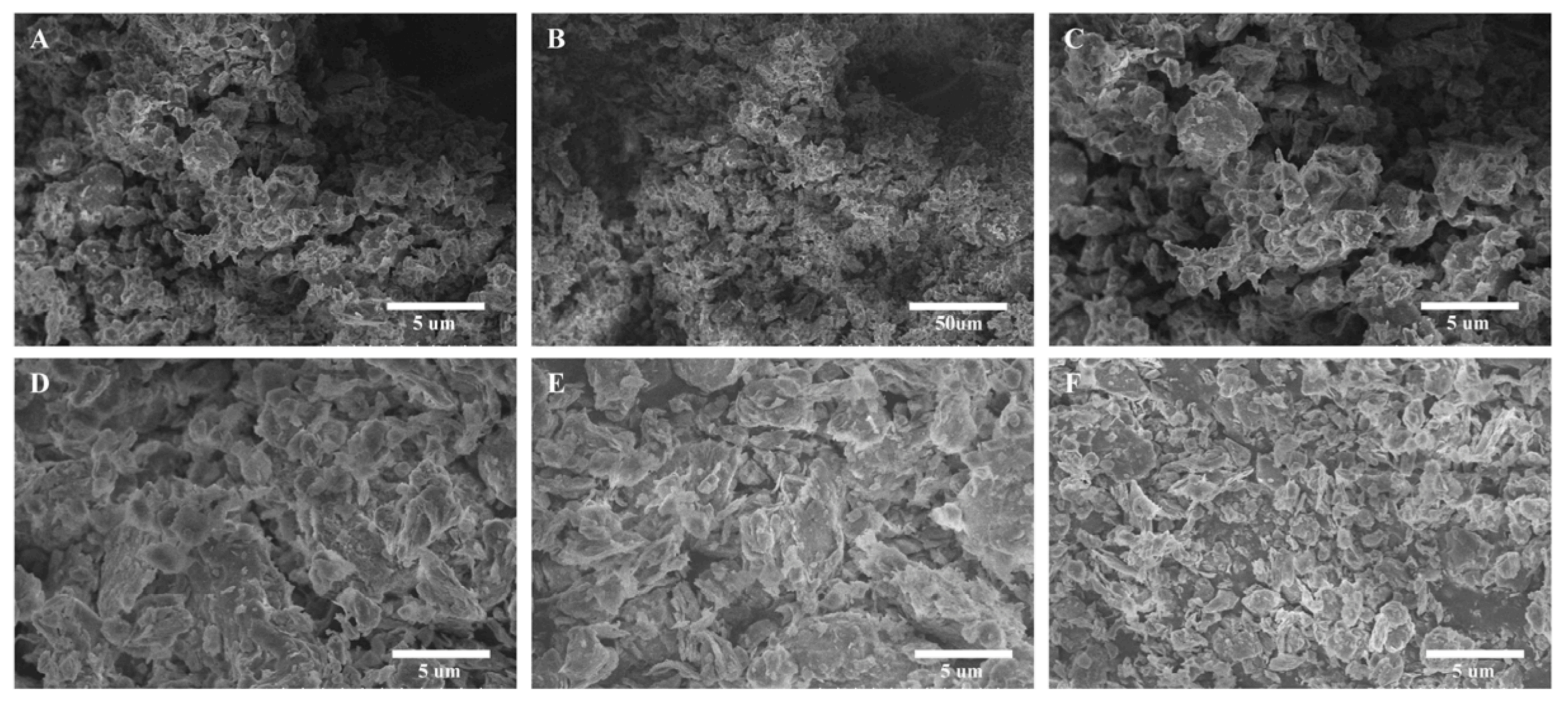
SEM images of (**A**,**B**) raw coal gangue and products calcined for 2 h at (**C**) 300, (**D**) 500, (**E**) 700, and (**F**) 900 °C. Lamellar raw particles (**A**,**B**) roughen at 300 °C (**C**), delaminate at 500 °C (**D**), develop mesoporous metakaolin at 700 °C (**E**), and sinter at 900 °C (**F**). Adapted from Zhang et al. [90], © 2025, MDPI, Basel, under CC BY 4.0.

The morphological evolution accompanying these transformations is illustrated in Figure 10. Raw coal gangue consists of compact, well-defined kaolinite lamellae with smooth surfaces (Figure 10A,B). After calcination at 300 °C, the particles remain essentially intact but develop mild surface roughening as physisorbed water is expelled (Figure 10C). At 500 °C the ordered sheets begin to delaminate and fragment (Figure 10D). At 700 °C, a well-developed, interconnected mesoporous texture appears and the specific surface area reaches a maximum (13.8 m^2^/g), reflecting the formation of highly reactive metakaolin (Figure 10E). By contrast, heating to 900 °C causes densification and partial pore collapse as the newly formed metakaolin sinters (Figure 10F). These SEM observations are used to illustrate morphology only; phase identification is based on the XRD, FTIR, and TGA–DSC evidence above rather than on texture alone.

The principal reactions summarized above are:

4FeS_2_ (pyrite) + 11O_2_ → 2Fe_2_O_3_ + 8SO_2_↑ (1)

Al_2_Si_2_O_5_(OH)_4_ (kaolinite) → Al_2_O_3_·2SiO_2_ (metakaolin) + 2H_2_O↑ (2)

3γ-Al_2_O_3_ + 2SiO_2_ (metastable) → 3Al_2_O_3_·2SiO_2_ (mullite) (3)

2(Al_2_O_3_·2SiO_2_) (metakaolin) → Al_4_Si_3_O_12_ (spinel) + SiO_2_ (amorphous silica) (4)

Within the 600–800 °C operative window, kaolinite dehydroxylation is essentially complete, forming amorphous, high-surface-area metakaolin with mixed-coordinated Al(IV)/Al(V). However, the optimal activation temperature is not universal. Calcination below 500 °C results in incomplete dehydroxylation (“under-burned” products), whereas excessively high temperatures (e.g., 900 °C) cause pore collapse and densification, and above 950 °C, metakaolin recrystallizes into poorly reactive mullite Equation (3) [12,88]. Holding time has a minor effect once dehydroxylation is complete [92].

### 4.3. Chemical Activation

Chemical activation, as used in this review, refers to alkaline dissolution or acid leaching pathways that disrupt the dense aluminosilicate network of calcined coal gangue so as to release target elements into solution [93]. It is important to distinguish this element-recovery function from alkali activation in the geopolymer/cementitious sense, in which dissolved aluminosilicate species polycondense into N–A–S–H and C–A–S–H gels to form cementitious products; the latter is a construction-material application and lies outside the scope of this review. Accordingly, this section treats NaOH and Na_2_CO_3_ strictly as dissolution reagents that convert Al- and Si-bearing phases into soluble sodium salts, and HCl and H_2_SO_4_ as acidic lixiviants that dissolve the target metals. The specific reactions, newly formed phases, solubility of products, and impurity behavior for each route are detailed below.

(1) Alkaline dissolution for element recovery

NaOH dissolution. Under hydrothermal conditions, OH^−^ attacks the bridging Si–O–Si and Si–O–Al bonds of metakaolin, breaking down the aluminosilicate framework and converting the active Al- and Si-bearing components into water-soluble sodium salts [94,95]. The dissolution of the active components can be expressed by the following reactions in Equations (5) and (6) [95]:

Al_2_O_3_ + 2NaOH + 3H_2_O → 2Na[Al(OH)_4_] (5)

SiO_2_ + 2NaOH → Na_2_SiO_3_ + H_2_O (6)

The dissolved sodium aluminate (Na[Al(OH)_4_]) and sodium silicate (Na_2_SiO_3_) are both water-soluble. In the presence of Ca-bearing minerals, Al initially precipitates as the sodalite-like phases Na_8_Al_6_Si_6_O_24_(OH)_2_(H_2_O)_2_ and Ca_2_Al_2_SiO_6_(OH)_2_. Under prolonged NaOH treatment, these intermediate phases subsequently convert to the alumina-free solid phase NaCaHSiO_4_ [93,94]. This conversion serves as a critical mechanism for Al–Si separation: it liberates Al into the solution while locking Si in the solid residue, thereby significantly enhancing Al selectivity [94]. Meanwhile, Fe and Mg impurities are largely precipitated as insoluble hydroxides at high pH and report to the solid residue [93].

Na_2_CO_3_ (soda) roasting. At 800–1000 °C, Na_2_CO_3_ reacts with the active Al_2_O_3_ and SiO_2_ of metakaolin as in Equations (7) and (8) [95,96]:

Al_2_O_3_ + Na_2_CO_3_ → 2NaAlO_2_ + CO_2_↑ (7)

SiO_2_ + Na_2_CO_3_ → Na_2_SiO_3_ + CO_2_↑ (8)

Both sodium aluminate and sodium silicate are water-soluble and report to the leachate. However, dissolved silica readily reprecipitates with aluminate as an insoluble sodium aluminosilicate hydrate upon leaching, so a desilication step is generally required [95]. This route preferentially solubilizes Al, whereas Fe, Ti and Ca remain as insoluble oxides or calcium silicates in the residue [95]. Furthermore, although Na_2_CO_3_ roasting achieves high Al extraction, its stoichiometric consumption is substantial. Guo et al. [97] demonstrated that co-roasting coal gangue with bauxite red mud (which contains Na_2_O) can partially substitute for Na_2_CO_3_, reducing its consumption from 100% to 12.1–20.5% while maintaining >90% Al_2_O_3_ extraction.

(2) Acid leaching.

HCl leaching. H^+^ dissolves the active Al_2_O_3_ and Fe_2_O_3_ of metakaolin, while quartz and amorphous silica largely remain as an insoluble residue as in Equations (9) and (10) [93,98]:

Al_2_O_3_ + 6HCl → 2AlCl_3_ + 3H_2_O (9)

Fe_2_O_3_ + 6HCl → 2FeCl_3_ + 3H_2_O (10)

AlCl_3_ and FeCl_3_ are both water-soluble, so Al and Fe co-dissolve and downstream purification (solvent extraction or precipitation) is required to separate Fe^3+^ from Al^3+^. The residual SiO_2_ is separated as silica slag [99].

H_2_SO_4_ leaching: Al_2_O_3_ and Fe_2_O_3_ are dissolved to water-soluble sulfates as in Equations (11) and (12) [98,99]:

Al_2_O_3_ + 3H_2_SO_4_ → Al_2_(SO_4_)_3_ + 3H_2_O (11)

Fe_2_O_3_ + 3H_2_SO_4_ → Fe_2_(SO_4_)_3_ + 3H_2_O (12)

Al_2_(SO_4_)_3_ and Fe_2_(SO_4_)_3_ are both water-soluble, and the Fe/Al separation issue noted for the HCl route applies here as well. When Ca-bearing minerals (e.g., calcite, anorthite) are present, gypsum (CaSO_4_·2H_2_O) forms as a poorly soluble byproduct that can coat particle surfaces and impede further leaching—an established disadvantage of sulfuric acid relative to hydrochloric acid [99].

In summary, chemical activation for element recovery—whether alkaline dissolution (NaOH, Na_2_CO_3_) or acid leaching (HCl, H_2_SO_4_)—offers high selectivity and controllability, but reagent cost and high-salinity wastewater treatment remain the principal barriers to large-scale application [97,98,99]. Cementitious alkali activation (geopolymerization), which is not directed at element recovery, is outside the scope of this review.

### 4.4. Microwave Activation

Microwave activation achieves inside-out volumetric heating through the coupling effect between electromagnetic waves and material molecules, thereby efficiently stimulating the pozzolanic activity of coal gangue at lower energy consumption [3,100]. The core mechanism lies in the fact that the microwave field drives the dipole rotation of polar water molecules and hydroxyl groups within the coal gangue, while simultaneously inducing intense thermal motion of ions such as Al^3+^ and Si^4+^, generating heat rapidly through internal friction. This process not only accelerates the dehydration and dehydroxylation of kaolinite but also effectively disrupts and reconstructs the Si–O and Al–O bonds within the mineral lattice, ultimately driving the phase transformation from kaolinite to metakaolin [3,100,101]. Therefore, the phase transformation process of coal gangue under microwave irradiation is essentially consistent with that of conventional heating methods.

The optimal temperature range for microwave activation differs significantly from that of conventional thermal activation. When the microwave activation temperature reaches 400 °C, coal gangue begins to exhibit a molten state. As the temperature rises to 500 °C, micro-particles start to form internally. This is primarily attributed to the high-temperature environment generated by microwave radiation, which significantly enhances the solubility of various mineral phases, promotes particle refinement, and results in a liquid-like wetting state [101]. Upon reaching 600 °C, the mineral grain boundaries are disrupted, leading to the formation of metakaolin [102]. Consequently, the optimal calcination temperature for microwave activation is approximately 600 °C, which is significantly lower than the mainstream range of 600–800 °C for conventional thermal activation. Under these conditions, the dehydroxylation degree of kaolinite in coal gangue reaches its maximum, and the reaction time is shorter, with overall energy consumption reduced by 25–30% compared to conventional calcination [3,100]. Su [103] found that metakaolin can be formed after microwave calcination at 540 °C for just 10 min, whereas conventional calcination requires heating at 750 °C for 120 min to achieve the same degree of activation. Further research indicates that the phase transformation rate of kaolinite under microwave irradiation is approximately 4 × 10^12^ times faster than that under conventional heating, with the phase transformation temperature reduced by about 200 °C [103].

Studies indicate that the high calcination efficiency of microwave activation is largely attributed to the strong microwave absorption capacity of the container [104,105]. However, due to the inherently weak microwave absorption capacity of coal gangue, it is difficult to achieve a rapid internal temperature rise within the microwave field [106]. Directly adding microwave absorbers to coal gangue can effectively improve calcination uniformity, which is conducive to large-scale production [107].

Although microwave activation of coal gangue has demonstrated significant application potential [108,109], its large-scale industrial application still faces numerous technical barriers, primarily including the design of large-scale microwave cavities, control over the uniformity of internal energy distribution, and the development of continuous production processes [3,100]. To overcome these barriers, emerging industrial pathways include continuous tunnel microwave kilns and the addition of microwave absorbers (e.g., residual coal) to improve heating uniformity.

### 4.5. Complex Activation

Complex activation technology couples two or more activation methods to leverage synergistic effects among multiple mechanisms, effectively overcoming the performance bottlenecks of single activation modes. It has now become a core research direction in the activation of coal gangue [3,10]. Typical composite activation pathways include mechano-thermal activation [80], mechanochemical activation [110], thermochemical activation [111], and tertiary mechano-thermochemical coupling activation [112,113]. Compared to single activation, composite activation processes demonstrate significant advantages in disrupting the kaolinite structure and enhancing the extraction efficiency of metal elements [111]. The underlying mechanism can be described as a sequential, energy-coupled process rather than as a demonstrated non-additive synergy:

(1) Mechanical pretreatment introduces lattice distortion, point defects, and particle-size reduction, increasing the specific surface area and exposing fresh, under-coordinated surfaces [81,82];
(2) Subsequent calcination then requires a lower dehydroxylation temperature because these defective kaolinite crystallites transform more readily, so reactive amorphous metakaolin is formed over a narrower heating window [78,89,90];
(3) Chemical activation of the freshly formed metakaolin proceeds more readily still, because the Al(VI) octahedra have already been converted to a more labile Al(IV) + Al(V) mixed coordination; activators such as Na_2_CO_3_, ammonium sulfate, or KOH therefore convert the inert Al–Si framework into soluble salts (e.g., sodium aluminate, water-soluble ammonium alum) at lower reagent dose [95,114];
(4) Water or acid leaching finally releases these soluble species into solution.

We note that although many studies report higher extraction yields under combined activation than under any single pretreatment, relatively few have quantitatively decomposed the combined effect into additive versus interactive contributions; therefore, we use the term ‘synergistic’ in the operational sense of coupled mechanisms, and we do not claim that the combined effect is universally greater than the sum of individual effects without quantitative evidence [3,111,115].

Table 5 summarizes representative performance parameters of the five activation technologies. We caution that the numerical values reported in the literature are obtained from different response variables and baselines and therefore cannot be ranked on a single common percentage scale. Mechanical and chemical activation are usually quantified by the leaching or extraction efficiency of a specific element, whereas thermal and microwave activation are more often assessed by the degree of dehydroxylation, the content of amorphous metakaolin, or pozzolanic activity. Because cementitious reactivity, elemental extraction, dissolution, and dehydroxylation endpoints are not interchangeable, Table 5 reports, for each method, the original endpoint actually used in the cited studies rather than imposing a uniform ‘reactivity enhancement’ metric.

## 5. Recovery and High-Value Utilization of Valuable Elements in Coal Gangue

### 5.1. Bulk Element Recovery and Valorization

#### 5.1.1. Aluminum Recovery and Valorization

The alumina (Al_2_O_3_) content in coal gangue typically ranges from 15% to 35%, making it the most abundant valuable metallic component. This content level is comparable to that of low-grade bauxite, positioning coal gangue as a potential alternative resource to bauxite [96].

In coal gangue, aluminum typically coexists with silicon, primarily in the form of kaolinite (Al_2_Si_2_O_5_(OH)_4_). Based on the Al_2_O_3_/SiO_2_ mass ratio (A/S ratio), coal gangue can be classified into three categories: A/S ratio ≥ 0.5, 0.3 < A/S ratio < 0.5, and A/S ratio ≤ 0.3 [99]. Coal gangue with an A/S ratio ≥ 0.5 is predominantly used for the preparation of zeolite molecular sieves. Coal gangue with an A/S ratio between 0.3 and 0.5, featuring moderate aluminum and silicon contents, serves as an ideal raw material for extracting Al and Si elements, enabling the production of various products such as alumina [116], aluminum chloride [110], sodium silicate, and silica [117,118]. In contrast, coal gangue with an A/S ratio ≤ 0.3 is typically utilized as a raw material for manufacturing construction materials, including cement, ceramic tiles, and split bricks [119]. These low-A/S and bulk-construction-material routes are noted for classification context only; the remainder of this review focuses on element recovery (Al, Fe, Li, Ga, REEs) from higher-A/S feedstocks, with silicon treated as a byproduct rather than a primary target.

Researchers both domestically and internationally have developed various methods for extracting aluminum from coal gangue, which can be broadly categorized into alkaline and acidic processes. Through these extraction processes, aluminum-based products such as aluminum hydroxide (Al(OH)_3_), alumina (Al_2_O_3_), and ammonium alum can typically be prepared [120].

##### Alkaline Process

The alkaline process primarily comprises the sintering process and the hydro-chemical process. The sintering process involves the co-sintering of limestone and coal gangue, with a workflow that includes material sintering, clinker self-grinding, alkaline dissolution, desilication, carbonation, and calcination. The fundamental principle of this method is to co-sinter coal gangue and limestone at high temperatures (1100–1200 °C). This triggers an activation reaction between the aluminum-bearing crystalline and amorphous phases in the gangue and the limestone. During this process, aluminum is converted into soluble calcium aluminate, while silicon forms insoluble dicalcium silicate, thereby achieving phase separation [9]. Subsequently, the activated clinker undergoes alkaline leaching with a sodium carbonate solution. The calcium aluminate dissolves and transforms into sodium aluminate, whereas the dicalcium silicate remains insoluble in both water and sodium carbonate solution, facilitating efficient aluminum-silicon separation [121]. Finally, alumina can be produced from the leaching solution through carbonation and calcination.

The alkaline sintering route proceeds through five balanced reactions.

At 1100–1200 °C, the alumina component of kaolinite reacts with limestone to form soluble calcium aluminate:

Al_2_O_3_ + CaCO_3_ → CaAl_2_O_4_ + CO_2_↑ (13)

simultaneously, silica forms insoluble dicalcium silicate:

SiO_2_ + 2CaCO_3_ → Ca_2_SiO_4_ + 2CO_2_↑ (14)

During aqueous leaching with Na_2_CO_3_ solution, calcium aluminate dissolves and reprecipitates Ca as carbonate:

CaAl_2_O_4_ + Na_2_CO_3_ + 4H_2_O → 2Na[Al(OH)_4_] + CaCO_3_↓ (15)

Carbonation decomposes the aluminate liquor:

2Na[Al(OH)_4_] + CO_2_ → 2Al(OH)_3_↓ + Na_2_CO_3_ + H_2_O (16)

Calcination of the hydroxide gives alumina:

2Al(OH)_3_ → Al_2_O_3_ + 3H_2_O↑ (17)

Reactions (13) and (14) occur in the solid state at 1100–1200 °C; reactions (15) and (16) occur in aqueous slurry at near-ambient temperature; reaction (17) occurs at 1000–1200 °C. The insoluble Ca_2_SiO_4_ reports to the residue, achieving Al–Si phase separation.

Generally, the sintering process requires feedstocks with a relatively high alumina-to-silica ratio, whereas this ratio in coal gangue is typically below 1 [122]. Furthermore, the sintering process not only generates substantial amounts of dicalcium silicate residues that hinder the synchronous recovery of silicon resources, but its high-temperature environment (1100–1200 °C) also results in significant energy consumption. More critically, the inevitable dissolution of silica in alkaline solutions necessitates an additional desilication step [122]. Constrained by these technical bottlenecks, the sintering method is unsuitable for large-scale industrial production [121].

Drawing on the traditional Bayer process, the hydro-chemical process employs alkaline wet leaching to convert the alumina in coal gangue into soluble sodium aluminate, thereby achieving the separation and recovery of aluminum [123]. However, when treating feedstocks with a low alumina-to-silica ratio, the dissolved silica readily reacts with alumina to form insoluble sodium aluminosilicate hydrate (sodium silicate slag) precipitates [97]. This not only leads to the loss of aluminum resources but also forces the introduction of complex desilication processes.

In summary, due to inherent limitations such as the difficulty of aluminum-silicon separation and the tendency for secondary precipitation, the application prospects of alkaline processes in the field of aluminum extraction from coal gangue are not optimistic.

##### Acidic Process

At the mechanistic level, acid leaching of calcined coal gangue is a proton-driven, interface-controlled breakdown of the amorphous metakaolin network rather than a simple bulk dissolution. After kaolinite dehydroxylation, the original Al(VI) octahedra are converted into a labile Al(IV) + Al(V) mixed coordination; H^+^ ions then attack the Al–O and bridging Si–O–Al linkages, protonating the bridging oxygens and cleaving them so that octahedrally hosted Al is preferentially released into solution as soluble Al^3+^ (AlCl_3_ or Al_2_(SO_4_)_3_), whereas the Si–O framework is only partially attacked and repolymerizes in situ into an amorphous, silica-rich residue or hydrated silica gel [124]. XRD of the leach residue shows a broad diffuse band near 20–25° (2θ) with the kaolinite/alumina reflections largely lost, confirming that most Al has been extracted while silica remains as the solid product layer [124]. This process follows the shrinking core model: kinetic modeling of HCl leaching demonstrates that it is controlled by the surface chemical reaction, with an apparent activation energy of 45.64 kJ/mol [124]. For H_2_SO_4_ leaching, the reaction is also controlled by interfacial chemical reaction, with an apparent activation energy of 48.97 kJ/mol [125]. In contrast, during HCl leaching of high-iron gangue, both Al and Fe releases follow a mixed-control model, with Al and Fe exhibiting nearly identical apparent activation energies (55.5 and 55.8 kJ/mol, respectively) [91], indicating that they are liberated through the same acid-attack step rather than through separate mechanisms. These co-dissolution kinetics directly govern reagent consumption and downstream purification. Because Fe-bearing (hematite, siderite), Ca-bearing (calcite, anorthite) and Mg-bearing (dolomite) phases consume H^+^ stoichiometrically alongside Al, every mole of co-dissolved Fe^3+^, Ca^2+^ and Mg^2+^ adds to acid consumption and enters the leachate as an impurity. In sulfate media, Ca^2+^ additionally precipitates as poorly soluble gypsum that can coat particle surfaces and impede further leaching [125]. The Fe^3+^ co-extracted with Al must then be removed by solvent extraction or selective precipitation before an alumina product of acceptable purity can be obtained, so that Al-over-Fe/Ca/Mg selectivity, rather than the single Al recovery percentage, is what ultimately determines process economics.

**Figure 11:**
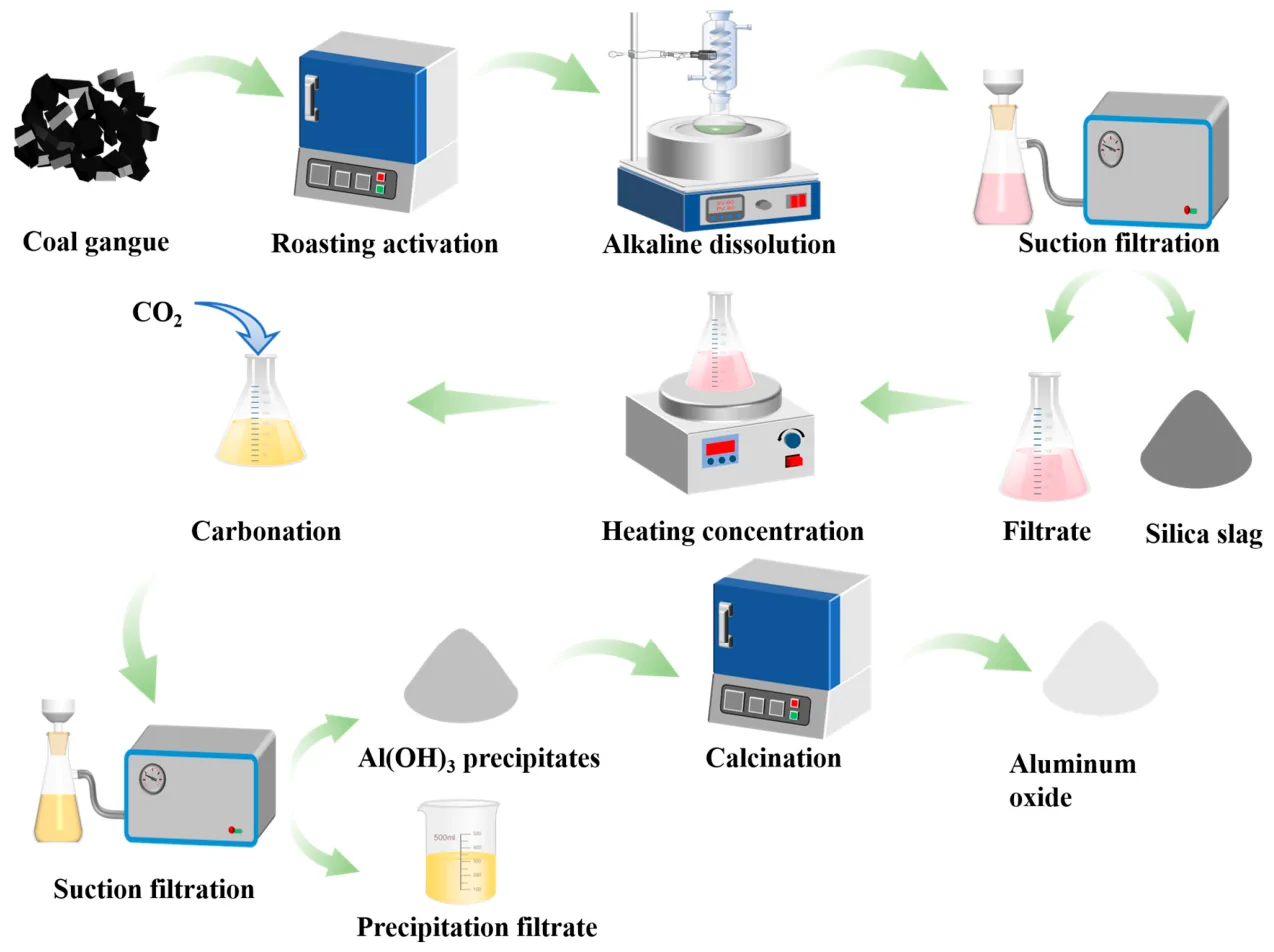
Flowchart of the sulfuric-acid leaching process for aluminum extraction from coal gangue.

The traditional “calcination–acid leaching” process typically achieves an alumina leaching efficiency of only 50–70% when treating coal gangue [22,98,126,127]. The overall sulfuric-acid leaching route is illustrated in Figure 11. To overcome this bottleneck, researchers have explored various strategies. Some scholars adopted a medium-temperature calcination combined with acid-pressure leaching process, which increased the alumina leaching efficiency to 80.19%; nevertheless, the improvement remains limited [128]. Furthermore, some studies have employed additives such as Na_2_CO_3_ to promote the transformation of kaolinite into metakaolin and enhance its reactivity, achieving alumina leaching efficiencies exceeding 90%. However, the high reagent costs severely hinder the widespread industrial application of this method [129,130,131].

In traditional acid leaching processes, the co-dissolution of various impurities alongside aluminum significantly complicates subsequent purification [132]. Furthermore, the inherent volatility, high cost, and strong corrosiveness of inorganic acids severely restrict their large-scale industrial application. To overcome these bottlenecks, Qin et al. [40] proposed a green ammonium salt roasting-water leaching process, achieving the synergistic extraction of aluminum and lithium with leaching efficiencies of 56.35% (Al) and 80.83% (Li), respectively. Although this process effectively avoids the use of strong acids or alkalis, there is still room for further improvement in the extraction efficiency of the target metals. Wang et al. [120] developed a novel “sulfuric acid roasting activation–water leaching–stirring crystallization” process for the preparation of NH_4_Al(SO_4_)_2_·12H_2_O. In this process, the aluminum leaching efficiency during the water leaching stage reached as high as 98.4%. Subsequently, ammonium sulfate was added to the leachate for crystallization, achieving an extent of Al crystallization (Al in crystals/Al in leachate) of 83.7% and a final product purity of up to 99.5%. However, the sulfuric acid roasting step in this process also faces severe equipment corrosion issues.

In summary, finding the optimal balance between leaching efficiency, equipment corrosion, and operational costs remains a critical technical challenge hindering the large-scale industrial application of the acid leaching process for aluminum extraction.

#### 5.1.2. Iron Recovery and Valorization

Iron in coal gangue primarily exists in the form of pyrite, siderite, and hematite [21,22]. Based on the iron oxide (Fe_2_O_3_) content, coal gangue can be classified into four categories: low-iron (0.1–1.0 wt.%), medium-iron (1.0–8.0 wt.%), high-iron (8.0–18.0 wt.%), and ultra-high-iron (>18.0 wt.%). Among these, high-iron and ultra-high-iron coal gangue possess significant value for iron extraction and serve as potential raw materials for producing iron oxide (Fe_2_O_3_) [9,21,22,133].

Currently, the mainstream processes for iron extraction from coal gangue mainly include reduction roasting–magnetic separation and acid leaching. Among these, the reduction roasting–magnetic separation method involves roasting coal gangue in a reducing atmosphere (e.g., H_2_, methane, or natural gas) to convert hematite into magnetite [134,135,136], followed by magnetic separation for recovery [117]. However, this process faces two major bottlenecks in practical application: first, the roasting temperature typically needs to reach 1100–1300 °C, resulting in extremely high energy consumption; second, some iron minerals remain in the form of intergrowths after roasting, making it difficult to achieve monomer dissociation through conventional magnetic separation, which limits the grade of the iron concentrate and the metal recovery rate.

Similar to aluminum extraction, the recovery of iron from coal gangue frequently employs a combined “roasting–acid leaching” process. For instance, Yang et al. [19] utilized thermal activation roasting combined with hydrochloric acid leaching, achieving an iron leaching rate of 87.64%, while SiO_2_ and most of the Al_2_O_3_ were retained in the solid residue. Kong et al. [91] further improved the iron leaching rate to over 90%. However, acid leaching processes inevitably cause equipment corrosion. To address this, researchers have proposed an (NH_4_)_2_SO_4_ roasting method to convert the iron-bearing phases (mainly hematite) in pre-calcined coal gangue into Fe_2_(SO_4_)_3_ [137]. Although this method requires only water washing to reduce the total iron content of the treated coal gangue to 0.08%, the addition of 40% (NH_4_)_2_SO_4_ during the roasting process results in high operating costs.

After purification, iron-rich leachates can be utilized to prepare iron oxide red pigments. Traditional purification processes typically induce iron precipitation by adjusting the pH to separate impurities. However, constrained by the complex metallic composition of the leachates, this method often faces bottlenecks such as stringent operating conditions, poor selectivity, and low separation efficiency, which restrict the purity of the final product [19]. For instance, the iron oxide red obtained by Zheng et al. [138] using this method had a purity of only 96.09%. In contrast, solvent extraction technology has been proven to efficiently separate and enrich iron from iron-rich leachates due to its advantages of high selectivity, high efficiency, and ease of continuous operation [139,140]. Among the various known extractants, tributyl phosphate (TBP) has demonstrated excellent extraction performance. Zhang et al. [141] found that a mixed system of TBP and methyl isobutyl ketone (MIBK) exhibits a significant synergistic effect on Fe^3+^ extraction, achieving a solvent-extraction recovery of up to 91.0%. Furthermore, Yang et al. [19] employed a TBP–sulfonated kerosene–hydrochloric acid system to selectively extract Fe^3+^, achieving an Fe purity of 99.21% among the co-extracted metal ions in the loaded organic phase. After stripping and hydrothermal conversion, the resulting iron oxide red reached a product purity of 99.16% (Fe_2_O_3_ mass fraction). Despite the remarkable effectiveness of solvent extraction, its high reagent consumption cannot be ignored. How to reduce operating costs while maintaining high extraction efficiency remains a critical technical challenge that needs to be addressed in future research.

In summary, high-purity iron oxide red can be produced from coal gangue through a sequence of roasting activation, acid leaching, solvent extraction purification, and hydrothermal synthesis. During the acid leaching stage for iron removal, iron reacts with hydrochloric acid to form ferric chloride, while some impurity ions, such as aluminum, magnesium, and calcium, also dissolve into the leachate Equations (18)–(23). Subsequently, in the solvent extraction purification stage, iron ions are selectively enriched, whereas impurities like aluminum, magnesium, and calcium are effectively removed. Finally, in the hydrothermal synthesis stage, sodium hydroxide solution is added to the iron-enriched solution, and the mixture is reacted in a hydrothermal autoclave Equations (22) and (23).

Al_2_O_3_·2SiO_2_ + 6HCl → 2AlCl_3_ + 2SiO_2_ + 3H_2_O (18)

Fe_2_O_3_ + 6HCl → 2FeCl_3_ + 3H_2_O (19)

MgO + 2HCl→MgCl_2_ + H_2_O (20)

CaO + 2HCl→CaCl_2_ + H_2_O (21)

FeCl_3_ + 3NaOH→Fe(OH)_3_ + 3NaCl (22)

Fe(OH)_3_ → Fe_2_O_3_ + 3H_2_O (23)

To address the bottlenecks of traditional processes in preparing iron oxide red, researchers have shifted their focus toward exploring new pathways for converting leached iron into high-performance functional materials. Du et al. [141] employed a combined “activation—acid leaching—solvent extraction–stripping” process to recover iron from high-iron coal gangue. Using the resulting stripping solution as an iron source, they successfully synthesized a zinc ferrite (ZnFe_2_O_4_) catalyst via hydrothermal and cetyltrimethylammonium bromide (CTAB)-assisted hydrothermal methods. Characterization results indicated that the catalyst possesses a pure crystalline phase structure and exhibits excellent catalytic degradation activity toward Rhodamine B (RhB). Furthermore, Du et al. [142] proposed a synergistic strategy integrating “coal gangue–chloride solution–electrodeposition–oxygen evolution reaction (OER) catalysis.” This approach enriches iron by regulating the chloride solution generated during coal gangue processing and subsequently electrodeposits it onto nickel foam (NF) to construct a high-performance iron-based OER electrode. These studies provide novel insights into the high-value utilization of iron resources from coal gangue.

### 5.2. Critical Trace Element Recovery and Valorization

This section is exclusively focused on three groups of critical trace elements: lithium (Li), gallium (Ga), and rare earth elements (REEs). These three groups were selected because they are the trace elements most extensively investigated for recovery from Chinese coal gangue and exhibit sufficient enrichment in specific coal-bearing strata to warrant dedicated process analysis. Other potentially valuable or critical elements occasionally reported in coal-derived wastes—such as vanadium (V), germanium (Ge), selenium (Se), and scandium (Sc)—are deliberately excluded from this section because they are predominantly enriched in coal combustion byproducts (e.g., fly ash) rather than in uncombusted coal gangue itself [143].

#### 5.2.1. Lithium Recovery and Valorization

##### Pre-Enrichment of Lithium

Due to the low lithium grade in coal gangue, direct extraction is economically unviable, making “pre-enrichment” particularly necessary. Studies indicate that the distribution of lithium in coal gangue exhibits significant particle size and density effects [32]. Chen et al. [39] pointed out that lithium concentration decreases with reduced particle size. In terms of density distribution, lithium is significantly enriched in medium-density fractions of 2.4–2.6 g/cm^3^ (e.g., kaolinite), a pattern that has also been corroborated by other researchers [48,144]. However, simple physical separation alone is insufficient to achieve efficient lithium enrichment. To address this, Fang et al. [33] conducted flotation tests using diesel and octanol as the collector and frother, respectively. They found that although most of the lithium was enriched in the tailings, its grade was only 1.02 times that of the raw ore, with a recovery rate of lithium-bearing minerals of only about 66%. Alternatively, Dai et al. [35] developed an intelligent sorting technology based on X-ray fluorescence (XRF) sensors. By identifying elemental content in real time and using electromagnetic ejection to discharge waste rock, this method increased the lithium grade in the concentrate from 100 ppm in the raw ore to 194 ppm and reduced the lithium concentration in the tailings to approximately 23 ppm; however, the yield of the concentrate was only 68.8%. In summary, research on the pre-enrichment of lithium from coal gangue is still in the exploratory stage, and there is an urgent need to develop more efficient and cost-effective sorting technologies.

##### Extraction of Lithium

Currently, technologies for lithium extraction from coal gangue remain in the research stage. The mainstream extraction processes primarily include direct leaching, mechanically assisted leaching, and roasting–leaching combined methods.

Because lithium is stably incorporated within the aluminosilicate lattice, direct extraction using acidic or alkaline solutions as lixiviants typically yields extremely low efficiencies, generally below 10%. For instance, studies by Chen et al. [39] and Zhang et al. [48] demonstrated that under acidic leaching conditions, lithium recoveries were merely 5.8% and 2.5%, respectively. In light of this, introducing an activation pretreatment step has become crucial for overcoming leaching bottlenecks and improving lithium recovery rates. Mechanical grinding, as an effective activation method, can disrupt the crystal structure of lithium-bearing minerals through mechanical forces, thereby significantly reducing the apparent activation energy required for the leaching reaction. Research by Fu et al. [145] indicated that following a 15-min ball milling pretreatment under optimal conditions, the lithium leaching efficiency could reach as high as 97.43%. However, the effectiveness of mechanical activation is not absolute; Chen et al. [32] reported that even when the grinding time was extended to 90 min, the maximum lithium leaching recovery was only 51.14%. This significant discrepancy suggests that the efficacy of the mechanical activation process is highly dependent on the specific physicochemical properties of the coal gangue and the microscopic occurrence modes of lithium within it.

The roasting–leaching process is currently recognized as the most effective technical pathway for recovering lithium from coal gangue. Multiple studies have confirmed the potential of this process: Wang et al. [29] achieved a lithium leaching rate of approximately 85% at an optimal calcination temperature of 500–600 °C; Zhang et al. [146] reached a 94% leaching rate by roasting slag at 400 °C for 20 min followed by acid leaching; and Xie et al. [147] obtained a peak Li leaching efficiency of 99.6% using sulfuric acid leaching after activation by roasting at 500 °C. However, despite the significant extraction efficiency of traditional inorganic acid lixiviants, issues such as severe equipment corrosion, high environmental pollution risks, and substantial alkali consumption for subsequent neutralization cannot be ignored. To address these challenges, Xie et al. [148] proposed a novel strategy for extracting lithium from coal-based lithium ores using recyclable organic acids. This approach not only achieved a 90% lithium recovery rate but also recovered over 90% of the acid via low-temperature recrystallization, significantly enhancing the greenness of the process. Furthermore, given that the acid leaching process inevitably dissolves impurity ions such as aluminum (Al) and iron (Fe), achieving selective lithium leaching is crucial. In this regard, Chen et al. [32] pretreated coal gangue via grinding-thermal activation and subsequently leached it with an ammonium sulfate solution. While achieving a lithium leaching rate of 84.76%, they successfully controlled the aluminum leaching rate at 17.63%, effectively improving the selectivity of the extraction process.

To address the bottleneck of high acid consumption in the roasting–acid leaching process, introducing chemical additives during the roasting activation stage to convert the target lithium components into water-soluble salts has emerged as an effective strategy. Wei et al. [49] utilized ammonium sulfate as an additive; under the conditions of a 1:2.5 mass ratio of coal gangue to ammonium sulfate and roasting at 450 °C for 2 h, they successfully converted lithium into soluble lithium ammonium sulfate, achieving a water leaching recovery of 88.35%. However, this process inevitably leads to the co-leaching of impurities such as aluminum and iron, significantly increasing the difficulty of subsequent separation and purification. To enhance leaching selectivity, Zhang et al. [23] proposed a novel method combining low-temperature roasting with potassium persulfate treatment, followed by water leaching. Under optimized conditions, the lithium leaching recovery reached 81.3%, while the leaching rates of aluminum (Al) and iron (Fe) were effectively controlled at 22.4% and 18.7%, respectively, thereby inhibiting impurity dissolution. Liu et al. [22] employed calcium chloride as an additive, mixing it with coal gangue at a mass ratio of 1:0.75 and roasting it at 800 °C for 2 h prior to acid leaching, which yielded a lithium recovery of 82.24%. Although these processes have improved lithium selectivity to some extent, they generally suffer from low extraction rates or excessive reagent consumption. Addressing this contradiction, Wang et al. [29] explored a technical pathway with reduced additive usage: adding 5% ferric sulfate to unroasted coal gangue for mechanical activation, followed by calcination at 600 °C. Under optimal leaching conditions (3 M HCl, 90 °C), this process achieved a lithium leaching rate of approximately 85%; however, this was accompanied by a significant increase in aluminum co-dissolution, which must be considered in downstream purification. Despite significantly reducing the consumption of chemical additives, there is still room for further improvement in the lithium leaching recovery of this process.

Beyond individual process descriptions, the variability in Li extraction efficiency across these studies can be systematically attributed to five controlling factors. (i) Li-hosting mineral: lithium hosted in clay minerals (kaolinite, illite) is more readily released by moderate activation than lithium incorporated in micas or resistant aluminosilicates, which explains the large discrepancy between the 97.43%97.43% [145] and 51.14%51.14% [32] reported under mechanical activation, as the efficacy of mechanical stress depends on the specific Li occurrence mode. (ii) Particle size and mechanical activation: grinding refines particle size and disrupts the crystal lattice, increasing surface area and active sites; however, excessive grinding induces agglomeration and cannot overcome lattice incorporation without thermal or chemical activation. (iii) Activation temperature: the roasting–leaching route exhibits a narrow optimal temperature window (typically 400–600 °C). At lower temperatures, dehydroxylation and phase transformation are incomplete; at higher temperatures (e.g., >800 °C), the formation of mullite or other inert phases encapsulates lithium, sharply reducing leachability [23,29]. (iv) Lixiviant type and concentration: strong inorganic acids (e.g., HCl, H_2_SO_4_) achieve high Li leaching but co-dissolve large amounts of Al and Fe, complicating downstream purification; ammonium salts and recyclable organic acids offer better selectivity but sometimes lower absolute leaching rates [32,49,148]. (v) Liquid-to-solid (L/S) ratio: increasing the L/S ratio generally improves leaching by lowering the slurry viscosity and enhancing mass transfer, but it also increases reagent consumption and dilutes the Li concentration, impacting downstream recovery economics. Optimizing these five factors synergistically, rather than maximizing any single variable, is essential for designing an industrial-scale Li extraction process.

##### Separation and Purification of Lithium

The leachate from coal gangue is characterized by a highly complex multi-component profile, making the development of highly selective lithium recovery technologies a critical step in the resource extraction process. Currently, the mainstream methods for lithium recovery primarily include precipitation, adsorption, and solvent extraction [149,150,151,152,153,154,155,156]. In the field of adsorption technology, Long et al. [151] prepared a Li_1.6_Mn_1.6_O_4_ precursor via microwave-assisted hydrothermal synthesis combined with solid-state calcination. Following acid washing, they successfully obtained an ion-sieve material Li_1.6_Mn_1.6_O_4_. Experimental results demonstrated that this material achieved a Li adsorption recovery of 99.9%. However, adsorbents often face bottlenecks such as poor structural stability and limited selectivity in acidic systems, which largely restricts their practical application in lithium extraction projects from coal gangue [152]. In the field of extraction technology, Wang et al. [156] developed a novel solvent extraction system using tributyl phosphate (TBP) as the extractant and sulfonated kerosene as the diluent. This system is capable of efficiently and selectively recovering Li^+^ from acidic coal gangue leachates, with a solvent-extraction recovery of 99.14%. Subsequently, the research team employed hydrochloric acid for a three-stage counter-current stripping process, achieving a stripping rate of 99.46%. Although this process has demonstrated excellent performance at the laboratory stage, given its relatively long flowsheet, its economic viability and feasibility for large-scale industrial application require further in-depth evaluation.

#### 5.2.2. Gallium Extraction and Valorization

##### Pre-Enrichment of Gallium

Compared to lithium, gallium is present in coal gangue at a significantly lower abundance; therefore, effective pre-enrichment is a critical step to ensure the purity and efficiency of subsequent extraction. Currently, exploratory studies have been conducted on the occurrence modes and pre-enrichment processes of gallium. Zhang et al. [48] observed through separation experiments that although gallium concentration tends to increase with larger particle sizes, conventional physical separation methods yield insignificant enrichment. Dai et al. [35] further elucidated the carrier mineral characteristics of gallium, identifying micas and clay minerals as the primary carriers, and noting a significant negative correlation between gallium and iron content. Based on these mineralogical properties, researchers attempted to utilize XRF sensor-based sorting technology to separate gallium concentrate from tailings. However, the improvement in the concentrate grade remains limited. Furthermore, Fang et al. [33] attempted flotation for pre-enrichment. The results showed that the gallium grade increased only slightly from an average of 30.5 ppm in the raw ore to 37 ppm, with a relatively low recovery rate of merely 66%. In summary, relying solely on screening or simple physical separation is insufficient for the efficient enrichment of gallium in coal gangue, highlighting an urgent need to develop novel and highly effective enrichment methods.

##### Extraction of Gallium

Similar to lithium, gallium in coal gangue is primarily hosted in aluminosilicate minerals, resulting in extremely low direct leaching efficiency [48]. Therefore, introducing specific activation pretreatment to disrupt the mineral lattice constraints is a critical prerequisite for the efficient hydrometallurgical recovery of gallium. Thermal activation roasting is currently the most widely used activation method. Studies indicate that during programmed heating, gallium in coal gangue gradually migrates from aluminosilicates to iron, potassium, magnesium, and calcium oxide components due to mineral phase transitions, and eventually returns to the aluminosilicate phase at 1000 °C [55]. The extraction efficiency of gallium exhibits a trend of initially increasing and then decreasing with rising roasting temperature [18,48], which is closely related to the transformation of kaolinite into mullite at high temperatures.

For thermally activated coal gangue, strong inorganic acids such as hydrochloric acid, sulfuric acid, and nitric acid serve as the primary leaching agents for gallium extraction. In terms of hydrochloric acid leaching, Zhang et al. [48] roasted coal gangue at 400 °C and subsequently leached it with 2 mol/L HCl at 60 °C for 4 h, achieving a gallium leaching efficiency of 54%. Similarly, Similarly, Aijie et al. [156] utilized hydrochloric acid and attained a 95% Ga leaching efficiency under optimized conditions (roasting at 600 °C for 0.5 h, followed by acid leaching with 6 M HCl at 100 °C for 6 h). This demonstrates that appropriately increasing the roasting temperature, acid concentration, leaching temperature, and leaching time is beneficial for enhancing gallium leaching efficiency. Regarding sulfuric acid leaching, Kang et al. [17] also achieved a gallium leaching efficiency of 95% under optimal conditions. Furthermore, Shao et al. [18] utilized nitric acid for autoclave leaching, reaching a maximum Ga leaching efficiency of 90%. Notably, using nitric acid as a leaching agent not only effectively dissolves valuable metals such as gallium, lithium, and aluminum but also retains iron in the solid residue, thereby facilitating subsequent separation and purification. Given the drawbacks of severe equipment corrosion and high costs associated with strong acid leaching, Qin et al. [113] proposed a non-traditional process combining potassium acetate intercalation with ammonium sulfate roasting. In this process, water leaching was performed after roasting, yielding a gallium leaching efficiency of 46%. Although this acid-free process offers a novel pathway for gallium extraction from coal gangue, its leaching efficiency still requires further improvement. Overall, research on gallium extraction from coal gangue remains in the exploratory stage.

##### Separation and Purification of Gallium

Gallium in leaching solutions is typically present at trace levels (ppm-level) and coexists with massive amounts of impurities such as aluminum and iron, posing significant challenges for separation and purification. Currently, the common methods for gallium extraction primarily include precipitation, solvent extraction, and adsorption. Regarding precipitation, Shi et al. [157] employed cupferron (C_6_H_9_N_3_O_2_) to selectively separate gallium and iron from simulated nitric acid leaching solutions of coal gangue and fly ash. Through parameter optimization, the Ga/Al separation factor reached as high as 2100, with a gallium recovery rate of 99%. In terms of solvent extraction, Zhao et al. [158] developed a closed-loop process for recovering gallium from sulfuric acid leaching solutions. The process first uses P507 as an extractant to remove impurities (Fe^3+^, Ti^4+^), with a gallium loss of only 1.2%. Subsequently, Cyanex 272 is used for one-step gallium extraction. After stripping with hydrochloric acid and washing with sulfuric acid, the extractant can be regenerated and recycled. This process demonstrates significant separation efficiency, with Fe/Ga and Ga/Al separation factors reaching 145 and 40, respectively. As for adsorption, Yang et al. [159] synthesized a novel high-performance polyurethane foam (PUF) adsorbent. Under optimal conditions, the gallium adsorption rate can reach 83.90%, and it maintains good performance even after multiple cycles. These studies provide valuable references for the efficient separation and enrichment of gallium from complex leaching solutions.

#### 5.2.3. Rare Earth Element Extraction and Valorization

Similar to the extraction of other valuable elements, the recovery of rare earth elements (REEs) typically requires roasting activation pretreatment [62].

Studies have shown that roasting can significantly enhance the TREE leaching efficiency, increasing it from approximately 20% to 90% [160]. Chen et al. [63] found that after calcination at 600 °C, rare earth minerals encapsulated within coal gangue particles are exposed. Meanwhile, bastnäsite (REFCO_3_) is converted into rare-earth oxides and rare-earth fluorides during thermal decomposition Equation (24), where RE denotes a trivalent light rare-earth element (predominantly Ce, La, Nd). Rare-earth oxides are more readily dissolved by acids, while the released fluoride ions form stable [AlF_6_]^3-^ complex ions with aluminum ions, further promoting the dissolution of rare-earth fluorides. Additionally, parisite (CaRE_2_(CO_3_)_3_F_2_) decomposes at approximately 540 °C into CaF_2_, RE_2_O_3_, and CO_2_ Equation (25). The roasting process can also effectively facilitate the enrichment of rare earth elements. Wu et al. [64] found that approximately 80% of the REEs in the calcined product exist primarily in the form of silicate-aluminosilicate components. Wang et al. [65] investigated the effectiveness of sodium carbonate and sodium chloride as roasting activators. Under optimal conditions, the leaching rates of REEs reached 91.41% and 68.88%, respectively. Meanwhile, the process effectively inhibited the dissolution of certain impurities. However, this method requires the consumption of large amounts of alkaline activators, generates significant residues, and leads to the co-dissolution of certain impurity ions with REEs, thereby increasing the complexity of subsequent extraction and separation processes.

3REFCO_3_(s) → RE_2_O_3_(s) + REF_3_(s) + 3CO_2_(g)↑ (400–600 °C) (24)

CaRE_2_(CO_3_)_3_F_2_(s) → CaF_2_(s) + RE_2_O_3_(s) + 3CO_2_(g)↑ (~540 °C) (25)

Acid leaching is a critical step in the extraction of REEs following calcination. Similar to the extraction processes for other metals discussed above, this process typically employs strong inorganic acids, such as hydrochloric, sulfuric, and nitric acids, as lixiviants. However, this approach poses significant challenges, including severe equipment corrosion and high costs. Compared to traditional inorganic acids, sulfamic acid, as a solid strong acid, offers distinct advantages, including low volatility and reduced corrosiveness. Zhu et al. [66] employed an alkali fusion–sulfamic acid leaching method to extract REEs from coal-series kaolinite. Under optimal conditions, the leaching efficiencies for total REEs (TREEs), light REEs (LREEs), and heavy REEs (HREEs) reached 84.91%, 88.43%, and 71.03%, respectively; the preferential recovery of LREEs over HREEs is consistent with the dominance of light REEs in coal-series strata [66]. However, such high-total-extraction routes are typically accompanied by substantial co-dissolution of Al and Fe, which increases downstream separation and purification loads. Addressing this trade-off, Wang et al. [62] proposed a mechanochemically assisted, ferric-sulfate-mediated route that achieves selective REE leaching. By combining calcination at 600 °C, addition of 5 wt% ferric sulfate, and 1 h of mechanochemical activation, this method achieved a 65% TREE leaching efficiency—lower in absolute terms than the 84.91% reported above—while the Al and Fe co-dissolution rates were suppressed to only 0.37% and 1.27%, respectively. Here, REE-over-impurity selectivity is defined as the ratio of REE leaching efficiency to the co-dissolution rate of the main impurity (Al or Fe); on this basis, the Wang et al. route exhibits markedly higher selectivity despite its lower absolute TREE recovery. As noted above, a lower total extraction may still represent superior overall process performance when impurity dissolution is substantially reduced, because downstream solvent-extraction and precipitation loads—and hence reagent consumption and purification costs—scale with the concentration of co-dissolved impurities rather than with the raw REE leaching percentage alone.

### 5.3. Silicon Recovery and Valorization

Although silicon is the dominant matrix component of coal gangue (SiO_2_ typically accounting for 35–55%, Table 1), it is not a primary target of the extraction routes reviewed above for Al, Fe, Li, Ga and REEs. Instead, silicon is recovered mainly as a byproduct after selective extraction of the valuable metals, either through desilication of the alkaline leachate or from the residue of acid leaching.

In the alkaline extraction route, dissolved silica readily reacts with aluminate to form insoluble sodium aluminosilicate hydrate, so that an additional desilication step is generally required [101]. Du et al. [161] proposed a stepwise strategy of “alkali activation-preferential silicon extraction-acid leaching for aluminum”: soda ash preferentially reacts with the silicon component at a controlled temperature and breaks the Si-Al bonds, after which high-purity liquid water glass (sodium silicate) is recovered by water leaching and the remaining aluminum-rich residue is subjected to hydrochloric acid leaching. Under optimized conditions, the leaching efficiencies of silicon and aluminum reached 82.1% and 92.36%, respectively, achieving efficient separation and high-value utilization of silicon.

The recovered silicon products can be further valorized into silica-based functional materials. Zhang et al. [162] extracted Al and Fe extracted from coal gangue were used to synthesize NiAlFe-LDHs, which were further calcined into NiAlFe-LDO layered oxides. The optimal LDO sample achieved ~96% degradation of rhodamine B within 20 min via peroxymonosulfate (PMS) activation. Meanwhile, the acid-leached SiO_2_ residue was reused as an inorganic support to fabricate NiAlFe-LDO/SiO_2_ composite, which exhibited ~95% adsorption efficiency toward tetracycline hydrochloride within 40 min, realizing the simultaneous valorization of both extracted metals and silica by-product. Yang et al. [163] synthesized a three-dimensional layered polysilicate-aluminum-ferric (PSAF) flocculant through a combined “alkali roasting–acid leaching–alkali dissolution” process, which exhibited good flocculation performance in coal preparation wastewater.

Nevertheless, the recovered silicon products are currently limited mainly to sodium silicate, silicon fertilizers, and other low-added-value additives; high-value applications such as high-purity silica and advanced silica-based functional materials remain at an exploratory stage. Silicon is therefore recovered mainly as a byproduct of metal extraction, and its high-value utilization warrants further study.

### 5.4. Synergistic Recovery and Valorization of Multiple Metals in Coal Gangue

Coal gangue contains various critical metal resources, including aluminum (Al), iron (Fe), silicon (Si), lithium (Li), gallium (Ga), and rare earth elements (REEs). However, traditional single-element extraction models often struggle to balance economic and environmental benefits, because maximizing the recovery of one target element frequently entails co-dissolution or immobilization of the others. Integrated multi-element recovery is one of the central questions of this review (review question 3 in the Introduction). Accordingly, the studies surveyed in this section are compared not on the basis of single-element maximum extraction percentages (as in Section 5.1 and Section 5.2), but on three flowsheet-level criteria: (i) whether the process recovers two or more target elements in compatible, stepwise or simultaneous stages; (ii) element-to-element selectivity—i.e., the relative leaching of target versus impurity elements; and (iii) downstream separability of the resulting pregnant solutions. As the following subsections show, the literature to date reports almost exclusively leaching-stage (or volatilization-stage) recoveries; complete flowsheet recoveries from run-of-mine gangue through to purified individual products remain scarce, and this data gap is itself a key finding of this review. In this regard, the academic community has nonetheless conducted numerous beneficial explorations.

From an economic perspective, comprehensive conversion is currently constrained by the high energy and reagent costs of processing bulk, low-value fractions; near-term viability therefore depends on co-extraction configurations (e.g., Al + Li or Al + Fe) that share these costs across multiple products.

#### 5.4.1. Synergistic Recovery and Valorization of Aluminum, Iron, and Silicon

To achieve the synergistic recovery of aluminum (Al) and iron (Fe) from coal gangue, Kong et al. [91] roasted coal gangue at 675 °C for 60 min, followed by leaching with 6 mol/L hydrochloric acid at 93 °C for 4 h. This process achieved leaching rates of 91% for iron and 66% for aluminum. However, due to the significant differences in the chemical activity of aluminum and iron components, the aluminum leaching efficiency is often low. Undissolved aluminum can react to form insoluble aluminosilicates during subsequent alkaline silicon extraction, which not only inhibits silicon dissolution but also wastes aluminum resources [93]. Furthermore, residual aluminum and iron impurities in the filter residue reduce the purity of silicon-containing products, restricting their high-value utilization [164]. Therefore, achieving efficient and synergistic leaching of aluminum and iron ions is crucial. To address this challenge, Kong et al. [165] proposed a calcium fluoride (CaF_2_)-assisted acid leaching process. Under optimized conditions with 3% CaF_2_ addition, the leaching efficiencies of Al^3+^ and Fe^3+^ were significantly improved from 62.96% and 85.12% to 92.10% and 95.73%, respectively. Based on this leachate, polymeric aluminum ferric chloride (PAFC) was successfully synthesized. Additionally, Zhang et al. [79] developed a method involving calcination activation, acid leaching, and reduction treatment of coal gangue to prepare an aluminum- and iron-enriched acidic solution and a silicon-enriched product. Under optimal conditions, the recovery rates of aluminum and iron reached 90.84% and 97.68%, respectively, while the silicon enrichment rate reached 83.90%.

To address the issues of insufficient purity and limited utilization of the silicon-rich residue following the aforementioned acid leaching process, Du et al. [161] proposed a stepwise extraction strategy of “alkali activation–preferential silicon extraction–acid leaching for aluminum.” This process utilizes soda ash to preferentially react with the silicon components in coal gangue at a specific temperature, effectively breaking the Si–Al chemical bonds. Subsequently, high-purity liquid water glass (sodium silicate) is separated via water leaching, and the remaining aluminum-rich residue is subjected to hydrochloric acid leaching to prepare a high-purity aluminum chloride solution. Under optimal conditions, the leaching efficiencies of silicon and aluminum reached 82.1% and 92.36%, respectively, achieving efficient separation and high-value utilization of silicon and aluminum in coal gangue.

Given the bottleneck that most coal gangue has a low iron content and poor economic viability for direct extraction, co-processing it with high-iron solid waste has become an effective pathway for cost reduction and efficiency improvement. Recent studies have made significant progress in this field: Zhao et al. [117] employed a co-roasting and sulfuric acid leaching process using coal gangue and coal gasification coarse slag. Under optimized conditions, this process achieved high leaching efficiencies of 79.93% for total iron (TFe) and 43.78% for Al^3+^, significantly reducing both energy and acid consumption. Additionally, Jin et al. [166] achieved the separation and recovery of iron and aluminum minerals through the co-roasting and magnetic separation of red mud and coal gangue. This process yielded an iron concentrate with a TFe grade of 57.25% (recovery rate of 65.22%) and an aluminum-enriched product with an Al_2_O_3_ content of 27.26% (recovery rate of 71.37%), realizing the efficient separation of iron-aluminum components and the valorization of solid waste. However, the overall leaching efficiencies in these attempts remain relatively low.

Researchers have explored the diversified high-value utilization of the separated Al, Fe, and Si components. Zhang et al. [162] extracted Al and Fe from coal gangue to synthesize NiAlFe-LDHs, which were calcined into layered oxides (NiAlFe-LDOs) that activated PMS and degraded about 96% of rhodamine B within 20 min. The acid-leached SiO_2_ residue was further used as a support to prepare NiAlFe-LDOs/SiO_2_, which adsorbed about 95% of tetracycline hydrochloride within 40 min, expanding the pathways for the resource utilization of coal gangue. Yang et al. [163] achieved the efficient separation of Si, Al, and Fe through a combined “alkali roasting–acid leaching–alkali dissolution” process. Utilizing the obtained components, they synthesized a polysilicate-aluminum-ferric (PSAF) flocculant with a three-dimensional layered structure. Experimental results indicated that this flocculant exhibits significant flocculation performance in coal preparation wastewater treatment.

#### 5.4.2. Synergistic Recovery of Gallium and Lithium

The synergistic recovery of lithium and gallium from coal gangue has attracted extensive research attention; however, existing processes generally face a bottleneck of low gallium recovery during simultaneous leaching. To address this issue, Wei et al. [49] proposed a novel process combining low-temperature ammonium sulfate activation with stepwise water/acid leaching. By extracting lithium via water leaching and gallium via acid leaching, they achieved the selective separation and synergistic extraction of both elements. Under optimal conditions, the recovery rates for lithium and gallium reached 88.35% and 90.28%, respectively, providing a green solution for the high-value utilization of coal gangue. Liu et al. [26] employed a method involving mixing coal gangue with calcium chloride (mass ratio of 1:0.75) and roasting at 800 °C for 2 h for activation. This was followed by stepwise leaching with 1 mol/L and 3 mol/L hydrochloric acid, achieving leaching rates of 82.84% for lithium and 90.99% for gallium, successfully realizing continuous extraction. Furthermore, Qin et al. [113] proposed a novel strategy of “pretreatment exfoliation coupled with cyclic roasting” to achieve the simultaneous extraction of Al, Li, and Ga from coal gangue. This process utilizes potassium acetate intercalation pretreatment to replace traditional high-energy grinding, significantly refining the particle size of the coal gangue and effectively exfoliating the layered silicate structure, thereby greatly enhancing reaction activity. During the subsequent ammonium salt roasting and water leaching, the leaching rates of Al, Li, and Ga reached 62.74%, 86.56%, and 46.18%, respectively, while effectively inhibiting the dissolution of Pb. Additionally, this process achieved full recovery of ammonia gas and ammonia-containing wastewater, reducing reagent costs while realizing the cyclic enrichment of lithium.

For the activation and extraction of lithium and gallium from coal gangue, Dai et al. [146] developed a rapid joule heating method. Under the optimal rapid joule heating conditions of 600 °C for 120 s, the leaching efficiencies of lithium and gallium reached 96.27% and 71.46%, respectively, significantly outperforming conventional thermal activation. Mechanism studies indicate that the instantaneous thermal shock generated by rapid joule heating induces intense dehydroxylation of kaolinite and muscovite, transforming them into amorphous metakaolin and dehydroxylated muscovite. This process substantially increases the specific surface area and pore development, facilitating the transformation of lithium and gallium hosted in aluminosilicate minerals from an acid-insoluble phase to an acid-soluble phase, thereby significantly enhancing leaching performance. Furthermore, Liu et al. [26] mixed coal gangue with sodium chloride and achieved volatilization rates of 81.62% for lithium and 95.47% for gallium under optimal rapid joule heating conditions of 1200 °C for 50 s. In summary, rapid joule heating demonstrates tremendous application potential for the recovery of lithium and gallium from coal gangue, owing to its distinct advantages of high efficiency, low energy consumption, and operational simplicity.

#### 5.4.3. Multi-Component Co-Extraction

Existing studies have primarily focused on the synergistic recovery of two to three metal elements with similar contents in coal gangue, while research on the co-extraction of all components remains relatively scarce. Kang et al. [17] employed a roasting activation-sulfuric acid leaching process to achieve the simultaneous leaching of Al, Li, Ga, and rare earth elements (REEs), with leaching rates reaching 86.51%, 74.46%, 95.45%, and 77.14%, respectively. The leaching behaviors of Al, Li, and Ga exhibited high consistency. Furthermore, characterization of the leaching residue revealed a silica content as high as 83.96% with a loose and porous structure, indicating its potential for the preparation of silicon-based materials. This study provides valuable insights into the comprehensive utilization of valuable metals in coal gangue. However, the traditional single-step acid leaching method lacks selectivity, often leading to the co-dissolution of multiple target elements and posing significant challenges for subsequent separation and purification. To address this, Wang et al. [54] proposed a stepwise leaching strategy based on the occurrence states and leaching kinetics of the elements. After calcination activation at 600 °C, the material was first leached at 50 °C for 15 min, selectively extracting over 80% of the REEs, while the loss rates of Ga and Li were only about 7%, and the leaching rates of Al and Fe were controlled at 2.94% and 2.21%, respectively. Subsequently, raising the temperature to 90 °C and extending the leaching time to 180 min allowed for the recovery of approximately 80% of Ga and Li. This process not only achieves the priority separation of REEs but also leaves the second-stage leachate rich in Ga, Li, and abundant Al resources, offering potential for further comprehensive development and high-value utilization.

## 6. Analysis and Outlook of Existing Recovery Technologies

### 6.1. Analysis of Existing Recovery Technologies

Valuable elements in coal gangue are predominantly hosted within the crystal lattices of clay minerals, such as kaolinite, in the form of isomorphic substitution. Due to their exceptional chemical stability, these elements are difficult to leach directly via conventional methods. Consequently, the “roasting activation + acid leaching” process has become the mainstream technology for extracting valuable metals. However, a closer examination of existing technological routes reveals significant technical bottlenecks and environmental risks.

First is the roasting pretreatment and activation stage. Studies indicate that the roasting activation temperature for coal gangue typically needs to be maintained between 600 °C and 800 °C, a process that consumes substantial thermal energy (primarily from natural gas or electricity). For low-grade coal gangue, thermal energy costs often account for 30% to 40% of total operating expenses. Furthermore, the risk of atmospheric pollution during high-temperature pretreatment cannot be ignored. Coal gangue is usually associated with pyrite and fluorine-bearing minerals. During roasting at 600 °C to 800 °C, these components are highly susceptible to conversion into acidic gases, such as sulfur dioxide and hydrogen fluoride, as well as fine particulate matter. If the flue gas purification system operates unstably or if there are defects in the process design, these harmful gases will directly threaten air quality and ecological safety around the plant, and may even induce biological toxicity effects.

Second is the hydrometallurgical extraction and separation/purification stage. Although traditional strong acid leaching processes achieve high extraction efficiencies, they generate substantial volumes of high-salinity, strongly acidic wastewater. This wastewater not only contains residual unreacted acid but is also enriched with impurity ions such as aluminum, iron, calcium, and magnesium, as well as trace heavy metals. Simultaneously, the neutralization and precipitation processes produce massive amounts of chemical gypsum and silica gel waste residues. These solid and liquid wastes have complex compositions and are difficult and costly to treat. If disposed of improperly, they can easily cause soil salinization and secondary contamination of groundwater systems, creating new environmental burdens. Additionally, coal gangue leachates have extremely complex compositions, containing high concentrations of impurity ions like aluminum, iron, calcium, and magnesium, while the concentrations of rare and scattered metals like lithium and gallium are relatively low. Recovering high-purity products necessitates complex processes such as multi-stage solvent extraction, ion exchange, or fractional precipitation, which significantly drives up the comprehensive costs of separation and purification.

In summary, establishing stringent “three wastes” (wastewater, waste gas, and solid waste) emission standards and equipping facilities with high-level pollution control measures constitute the bottom line for the survival and compliance of this industry. Future research and engineering practices should be dedicated to green innovation at the process source, fundamentally breaking the dilemma of high energy consumption and severe pollution.

### 6.2. Outlook

As the largest solid waste generated by the coal industry, the resource utilization of coal gangue has transcended the mere scope of waste disposal, evolving into a strategic issue concerning national energy and resource security, ecological environmental protection, and the realization of “dual carbon” goals. Although significant progress has been made in process optimization and material preparation, achieving large-scale, high-value, and clean utilization of coal gangue still faces numerous technical bottlenecks and challenges. Based on an analysis of current technical limitations and future industrial demands, the following prospects for future research directions are proposed:

(1) Coal gangue exhibits significant fluctuations in mineral composition and poor material homogeneity. The traditional “one-size-fits-all” treatment approach must be abandoned in favor of precision utilization based on mineralogical characteristics. By leveraging technologies such as Mineral Liberation Analyzer (MLA), Quantitative Evaluation of Minerals by Scanning Electron Microscopy (QEMSCAN), and big data analytics, the occurrence states of valuable elements can be accurately resolved. Furthermore, targeted activation processes tailored to the specific characteristics of coal gangue should be developed to overcome the challenges of low-temperature reconstruction, thereby achieving the selective dissociation of valuable components.
(2) Conventional acid and alkali leaching processes are characterized by high reagent consumption, severe equipment corrosion, and a high risk of secondary pollution. Green solvents, such as ionic liquids and deep eutectic solvents, hold great potential for the selective leaching of rare and scattered metals; however, challenges regarding cost, viscosity, and recycling must be urgently addressed. Additionally, the development of highly selective separation materials, such as molecularly imprinted polymers, is essential to construct multi-element cascade enrichment processes for aluminum, iron, lithium, gallium, and rare earth elements, ultimately enhancing resource recovery rates.
(3) Achieving total consumption of tailings is imperative to mitigate the risks associated with stockpiling. The utilization of tailings should be upgraded from ordinary construction materials to high-value-added functional materials, such as zeolite molecular sieves, aerogels, and nano-silica. Furthermore, establishing a circular industrial system integrating “coal-power-chemical-materials” by coupling processes like waste heat roasting, leaching, and underground backfilling will optimize material and energy cycles, thereby reducing production costs. Furthermore, source reduction during new mine development—such as XRF-based pre-separation to reject pyrite-rich fractions and backfilling gangue into mined-out voids—can fundamentally lower the long-term environmental burden.
(4) A comprehensive life cycle assessment (LCA) system for coal gangue resource utilization must be improved by establishing a full-process LCA database to quantify carbon and water footprints and identify critical environmental hotspots. Incorporating carbon benefits into economic evaluations will help propel the industry’s transformation from a purely “policy-driven” model to a dual-drive model powered by both market forces and green development.

## 7. Conclusions

Coal gangue is not only the largest source of solid waste in the coal industry but also a strategic “misplaced resource” rich in Al, Si, Fe, Li, Ga, and REEs. Its high-value utilization can yield energy-saving and emission-reduction benefits on a life-cycle scale—provided that the system boundary includes upstream calcination energy, reagent manufacture (acid, alkali, extractant), neutralization, wastewater treatment, and residue disposal, rather than being limited to the laboratory-scale leaching step. On a cradle-to-gate basis, these benefits remain unquantified because full LCA data are currently scarce—but they also mitigate the risks of gangue pile spontaneous combustion and water pollution at the source, demonstrating tremendous dual economic and ecological benefits. However, current industrialization technologies still face bottlenecks such as high energy and reagent consumption, as well as significant risks of secondary pollution. In light of this, future research urgently needs to achieve breakthroughs in key technologies, including low-temperature green activation, high-efficiency medium separation, and near-zero-emission closed-loop processes. Relying on Life Cycle Assessment (LCA) and carbon footprint tracking mechanisms, the industry can be driven to leap from “low-end extensive consumption” to “precision extraction and high-end manufacturing,” ultimately achieving a synergistic symbiosis of resource development, economic growth, and ecological protection. It is worth noting that silicon is currently recovered mainly as a byproduct of metal extraction, and its high-value utilization remains exploratory and warrants further study.

## Figures and Tables

**Table 1:** Chemical composition (wt.%, by XRF) of coal gangue from different regions in China.

Compositions	SiO_2_ (%)	Al_2_O_3_ (%)	Fe_2_O_3_ (%)	SO_3_ (%)	CaO (%)	MgO (%)	K_2_O (%)	Na_2_O (%)	TiO_2_ (%)
Heidaigou Coal Mine [23]	35.1	31	0.58	—	1.11	0.08	0.15	0.05	0.58
Wanfu mining area [24]	39.55	32.15	3.21	6.26	2.83	0.65	1.19	1.19	1.44
Gaotouyao Coal Mine [25]	53.42	28.21	7.32	2.83	1.98	1.28	3.18	0.63	1.15
Coal mine in Shuozhou City, Shanxi Province [26]	47.85	22.78	15.99	—	3.04	2.12	2.42	1.44	—
A mining area in Yunnan Province	53.99	24.85	7.98	6.61	1.03	1.55	2.35	0.24	1.2
Mining Area No.1 in Guizhou Province	39.8	19.98	17.81	2.94	10.6	1.12	1.44	0.47	4.89
Mining Area No.2 in Guizhou Province	46.42	18.08	17.32	1.2	6.18	2	2.19	0.25	5.42

Note: Oxide contents are weight percent (wt.%) as reported in the cited primary studies, determined by X-ray fluorescence (XRF) spectrometry; values may be on an as-received or ignited basis as stated by the original authors. The coal gangue samples from Yunnan and Guizhou provinces were analyzed by the authors’ team.

**Table 2:** Lithium content in coal gangue in China.

Source	Concentration, ppm	Bearing Minerals	Ref.
Zhungeer mining area, Inner Mongolia	310	Silicates	[29]
Heidaigou Coal Mine, Inner Mongolia	395.36	Kaolinite	[23]
coal mine in Shuozhou City, Shanxi Province	136.51	Silicates	[26]
coal mine in Guizhou Province	331	Kaolinite	[30]
Xian’an coalfield, Guangxi Province	550	Aluminosilicates	[31]
Jincheng coal seam, Shanxi Province	277	Clay minerals	[17]
Thermal coal preparation plant, Jungar, Inner Mongolia	223	Kaolinite	[32]
Pingshuo coal mine, Shanxi Province	344	Kaolinite	[33]

**Table 3:** Gallium content in coal gangue in China.

Source	Concentration, ppm	Bearing Minerals	Ref.
Heidaigou Coal Mine, Inner Mongolia	33.73	Kaolinite	[22]
coal mine in Shuozhou City, Shanxi Province	21.01	Kaolinite, boehmite, and calcite	[26]
Coal washing plant in the Zhungeer mining area, Inner Mongolia	50.1	Silicate	[54]
Xing’an League, Inner Mongolia	85–95	Calcite, aluminosilicate	[44]
Antaibao Mine, Shanxi Province	25	Silicate, sulfide	[35]
Jincheng seam, Shanxi Province	22	Silicate, aluminosilicate	[17]
Shuozhou, Shanxi province	38.82	Kaolinite	[53]

**Table 4:** Rare earth element (REE) content in coal gangue in China.

Source	Concentration, ppm	Bearing Minerals	Ref.
Jungar mining area, Inner Mongolia	259.13	No data	[62]
Haerwusu open-pit mine, Jungar coalfield, Inner Mongolia	449.2	Monazite, bastnaesite	[63]
Three different power plants in China	132.75	Silicate, aluminosilicate	[64]
Jinsha County, Guizhou Province	1013.02	Kaolinite	[65]
Shanxi Province	590.51	quartz, dickite, kaolinite	[66]
Shuozhou, Shanxi province	381	Silicate	[63]

**Table 5:** Representative response variables, reported endpoints, and demonstrated scales of five coal-gangue activation technologies.

Method	Measured Response Variable	Reference Condition & Typical Value	Feedstock/Application	Demonstrated Scale/TRL	Ref.
Mechanical	Ion leaching rate (Ca^2+^, Mg^2+^, K^+^) as mineralization potential indicator	Un-milled vs. milled for 4 h: Ca^2+^ 16.98% → 31.29%; Mg^2+^ 8.27% → 20.19%; K^+^ 0.53% → 3.54%	China (SiO_2_ 59.16%, Al_2_O_3_ 24.15%)	Lab scale (150 g/batch); TRL 3–4	[115]
Thermal	Dehydroxylation/amorphous metakaolin content (pozzolanic reactivity)	Characteristic kaolinite peaks disappear above 750–800 °C; amorphous hump confirmed by XRD/FTIR	Kaolinitic gangue	Lab scale (5 s suspension calcination); TRL 4	[89,90]
Chemical	Al extraction efficiency (leaching)	Up to 94.68% under optimal NaOH hydrothermal conditions	Alumina-rich coal gangue (Al_2_O_3_ 44.93%)	Lab scale (autoclave); TRL 3–4	[96]
Microwave	28 d compressive and flexural strength of mortar	Compressive +72.5%; flexural +42.32% (at 600–700 °C, MCG3 group)	Ordos kaolinite rock	Lab scale (45 g/batch); TRL 3	[101]
Complex	28 d compressive strength; activity index	55.4 MPa; activity index 112.04% (with 8 wt% CaCl_2_)	Bulangou mining area, Inner Mongolia	Lab scale (mortar specimens); TRL 4	[107]

The table reports the original endpoint actually measured in each cited study rather than a normalized “reactivity enhancement” percentage. Because the response variables differ fundamentally—ion leaching potential (mechanical), dehydroxylation (thermal), elemental extraction (chemical), mortar strength (microwave), and activity index (complex)—the numerical values are not directly comparable across rows and must not be ranked on a single scale. TRL follows a simplified convention: Laboratory = TRL 3–4 (bench-scale, gram- to kilogram batches); Pilot = TRL 5–6 (tonne-scale pilot); Mature = TRL 7–9 (industrial scale).

## Data Availability

All data supporting the findings of this review are included within the article and its cited references.
